# Intron retention and nuclear loss of SFPQ are molecular hallmarks of ALS

**DOI:** 10.1038/s41467-018-04373-8

**Published:** 2018-05-22

**Authors:** Raphaelle Luisier, Giulia E. Tyzack, Claire E. Hall, Jamie S. Mitchell, Helen Devine, Doaa M. Taha, Bilal Malik, Ione Meyer, Linda Greensmith, Jia Newcombe, Jernej Ule, Nicholas M. Luscombe, Rickie Patani

**Affiliations:** 1 The Francis Crick Institute, 1 Midland Road, London, NW1 1AT UK; 2 Department of Molecular Neuroscience, UCL Institute of Neurology, Queen Square, London, WC1N 3BG UK; 3 Sobell Department of Motor Neuroscience and Movement Disorders, UCL Institute of Neurology, Queen Square, London, WC1N 3BG UK; 4 Department of Neuroinflammation, UCL Institute of Neurology, Queen Square, London, WC1N 1PJ UK; 5 UCL Genetics Institute, University College London, Gower Street, London, WC1E 6BT UK; 6 Okinawa Institute of Science & Technology Graduate University, Okinawa, 904-0495 Japan

## Abstract

Mutations causing amyotrophic lateral sclerosis (ALS) strongly implicate ubiquitously expressed regulators of RNA processing. To understand the molecular impact of ALS-causing mutations on neuronal development and disease, we analysed transcriptomes during in vitro differentiation of motor neurons (MNs) from human control and patient-specific VCP mutant induced-pluripotent stem cells (iPSCs). We identify increased intron retention (IR) as a dominant feature of the splicing programme during early neural differentiation. Importantly, IR occurs prematurely in VCP mutant cultures compared with control counterparts. These aberrant IR events are also seen in independent RNAseq data sets from SOD1- and FUS-mutant MNs. The most significant IR is seen in the SFPQ transcript. The SFPQ protein binds extensively to its retained intron, exhibits lower nuclear abundance in VCP mutant cultures and is lost from nuclei of MNs in mouse models and human sporadic ALS. Collectively, we demonstrate SFPQ IR and nuclear loss as molecular hallmarks of familial and sporadic ALS.

## Abstract (web summary)

Intron retention (IR) can increase protein diversity and function, and yet unregulated IR may be detrimental to cellular health. This study shows that aberrant IR occurs in ALS and finds nuclear loss of an RNA-binding protein called SFPQ as a new molecular hallmark in this devastating condition.

## Introduction

Amyotrophic lateral sclerosis (ALS) is an age-related rapidly progressive and incurable condition, which leads to selective degeneration of motor neurons (MNs). ALS-causing mutations implicate crucial regulators of RNA processing—normally expressed throughout development—in the underlying pathogenesis. This raises the possibility that post-transcriptional changes occurring at early stages of life, including neurodevelopment, may play a pivotal role in the underlying molecular pathogenesis of ALS. Understanding the impact of ALS-causing mutations on neurodevelopment and disease will allow us to elucidate initiating molecular events. This in turn may guide the development of new therapies targeting primary mechanisms before the disease progresses too far.

Both development and homoeostasis of neurons fundamentally rely on the precise implementation of cell-type and stage-specific alternative RNA processing, including alternative splicing (AS) and alternative polyadenylation (APA)^1–3^. Several mechanisms of AS are established including exon-skipping, mutually exclusive exons and intron retention (IR). Alternative exon usage features particularly in neurons to regulate protein diversity and function^4^. Neural cell types exhibit a higher proportion of retained introns compared with other tissues and there is an expanding body of evidence demonstrating a functional role for IR in neuronal development and homoeostasis^5–8^. Increased IR during neuronal differentiation has been shown to downregulate the expression of transcripts that are unnecessary for mature neuronal physiology^7^. A recent study showed evidence for post-transcriptional processing of intron-retaining transcripts in response to neuronal activity^8^.

APA is an alternative mode of RNA processing that generates distinct 3′ termini most frequently in the 3′ untranslated region (3′ UTR) of mRNA, thereby engendering isoforms of variable 3′ UTR length. 3′ UTRs serve as a key platform in the RNA regulatory network controlling mRNA translational efficiency, localisation and stability^9,10^. 3′ UTRs harbour extensive tissue-specific length variability that significantly affect their function^11^. Of all human cell-types, brain-specific isoforms have the longest 3′ UTRs^12,13^. Moreover, mutations perturbing the mRNA secondary structure and sites of mRNA–miRNA interactions can lead to neurodegeneration^14^. Despite these findings, the roles of IR and 3′ UTR regulation in the context of MN development and homoeostasis have remained understudied compared with other forms of AS.

AS and APA are coordinated by the action of individual or combinations of trans-acting RNA-binding proteins (RBPs) that bind to specific sites within (pre-) mRNA^15,16^. RBPs mediate mRNA splicing, nuclear export and localisation. Accumulating evidence implicates RBPs as key regulators of both neurodevelopment and specific forms of neurodegeneration. Indeed, mutations in several RBPs including Transactive-response DNA-binding Protein, 43 kDa (TDP-43), Fused in Sarcoma (FUS) and TATA-Box Binding Protein Associated Factor 15 (TAF15) have been causally linked to familial form of ALS leading to RNA processing defects in mouse models^17,18^.

We hypothesised that aberrant splicing and APA play important roles in both development and disease of the nervous system. Against this background we sought to understand how different modes of AS and APA feature in human motor neurogenesis and to examine systematically the impact of ALS-causing mutations on post-transcriptional remodelling during lineage restriction. By combining directed differentiation of human-induced-pluripotent stem cells (iPSCs) with time-resolved RNA sequencing, we first identified the sequential programme of post-transcriptional remodelling that underlies human motor neurogenesis. We recently identified clear cellular phenotypes of ALS using valosin containing protein (VCP)-related patient-derived iPSCs^19^. This platform allowed us to compare VCP mutant (*VCP*^*mu*^) to control cultures during MN differentiation and identify mutation-dependent splicing deregulation in a developmental stage-specific manner. We identify Splicing Factor Proline and Glutamine rich (SFPQ) as the most significant intron-retaining transcript across diverse ALS-causing mutations (VCP, SOD1 and FUS). We show that the SFPQ protein binds extensively to its retained intron, which exhibits high cytoplasmic abundance in *VCP*^*mu*^ compared with controls. Crucially, the protein is less abundant in the nuclei of *VCP*^*mu*^ cultures and is ultimately lost from nuclei of MNs in mouse models (*SOD1*^*mu*^ and *VCP*^*mu*^ transgenic mouse models) and human sporadic ALS post-mortem samples. In summary, our study implicates SFPQ IR and nuclear loss as general molecular hallmarks of familial and sporadic ALS.

## Results

### IR is the predominant mode of splicing in motor neurogenesis

**Fig. 1:**
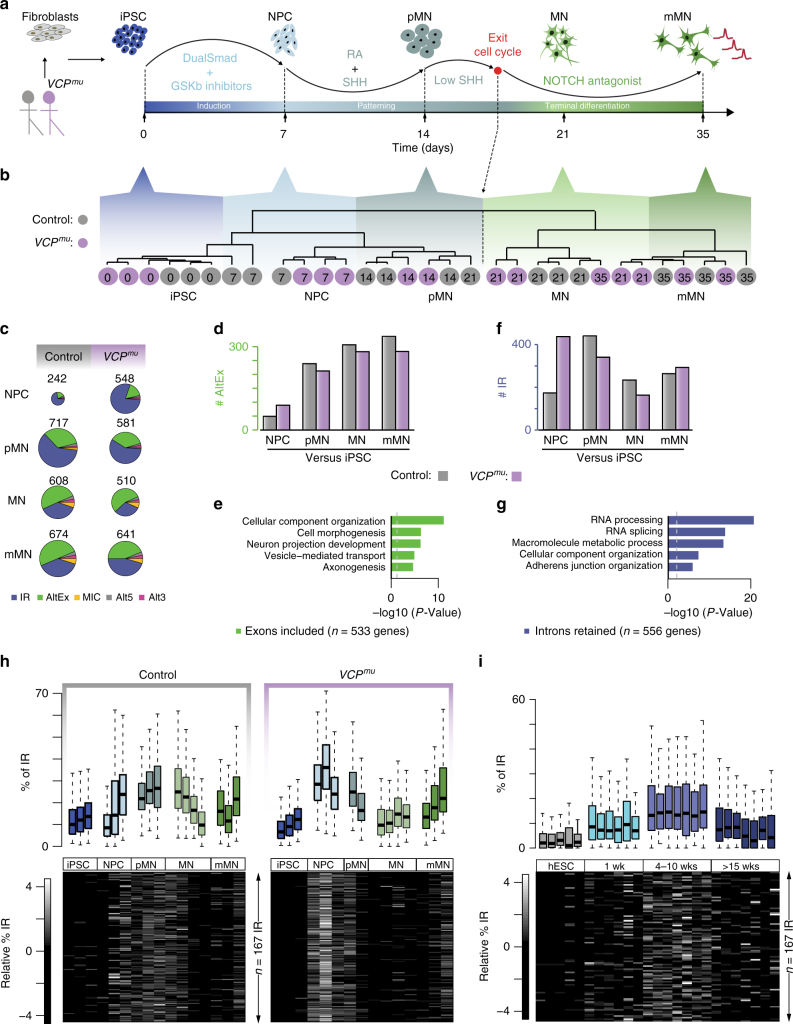
Intron retention is the predominant splicing change during early motor neurogenesis and occurs prematurely in *VCP*^*mu*^ cultures. **a** Schematic depicting the iPSC differentiation strategy for motor neurogenesis. Arrows indicate sampling time-points in days. iPSC clones were obtained from two patients with confirmed VCP mutations (R155C and R191Q; total 3 iPSC lines, 1 induction from each line) and 1 clone from each of 2 healthy controls (total 2 different iPSC lines, 2 inductions from one line and 1 induction from the other line). Induced-pluripotent stem cells (iPSC); neural precursors (NPC); “patterned” precursor motor neurons (ventral spinal cord; pMN); post-mitotic but electrophysiologically immature motor neurons (MN); electrophysiologically mature MNs (mMN). **b** Unsupervised hierarchical clustering of 15,989 genes groups the 31 samples according to neuronal developmental stage, rather than genetic background. Grey circles = control samples; magenta circles = *VCP*^*mu*^ samples; sampling time-points are indicated inside the circles. **c** Pie charts representing proportions of splicing events in control and *VCP*^*mu*^ samples at distinct stages of motor neurogenesis compared with the previous time-point. Chart areas are in proportion to total numbers of events at each stage. Intron retention (IR); alternative exon (AltEx); microexons (MIC); alternative 5′ and 3′ UTR (Alt5 and Alt3). **d**, **f** Bar graphs representing the numbers of exonic and intronic splicing events, respectively, in control (grey bars) and *VCP*^*mu*^ samples (magenta bars) at specific timepoints during MN differentiation. **e**, **g** Bar graphs showing the enrichment score of GO biological pathways associated with transcripts undergoing exonic and intronic splicing events in control samples. **h** Upper, boxplots depicting the distributions of percentage retention (see Methods) for 167 manually curated introns in replicates at distinct stages of differentiation in control (left) and *VCP*^*mu*^ samples (right). Boxplots display the five number summary of median, lower and upper quartiles, minimum and maximum values. Lower, heatmaps of the standardised relative percentage of IR in 167 introns in replicate samples at each differentiation stage. **i** As in **h** but for in vitro differentiation of hESCs to the neural induction stage (1 week), NPC stage (4–10 weeks) that produces only neurons upon further differentiation and after >15 weeks, a more gliogenic stage which produces both neurons and glial cells^22^

To examine post-transcriptional changes during human motor neurogenesis, we analysed high-throughput RNA-sequencing (RNA-seq) data for polyadenylated RNA isolated from induced-pluripotent stem cells (iPSCs; day 0), neural precursors (NPCs; day 7), “patterned” precursor MNs (ventral spinal cord; pMNs; day 14), post-mitotic but electrophysiologically immature MNs (MNs; day 21), and electrophysiologically mature MNs (mMNs; day 35) derived from two patients with the ALS-causing VCP gene mutation and two healthy controls (Fig. 1a; 31 samples from 5 time-points and 3 genotypes; 2 clones from 2 healthy controls and 3 clones from 2 ALS patients with VCP mutations: R155C and R191Q). Cellular samples from each stage of MN differentiation were characterised as previously reported^19^. Here, we conducted additional extensive characterisation to confirm highly enriched MN cultures (>90%; Supplementary Fig. 1a). Importantly, the MN differentiation efficiency was similar between control and VCP mutant cultures (Supplementary Fig. 1b,c). Using a set of 19 key gene markers of spinal MN maturation and embryonic development, we further confirmed a prior finding that iPSC-derived mMNs resemble foetal rather than adult MNs^20^ (Supplementary Fig. 2a,b). Unsupervised hierarchical clustering (Spearman rank correlation and complete clustering) of the 31 samples using 15,989 reliably expressed genes segregated samples based on their developmental stage within the MN lineage rather than by the mutant or control genetic background (Fig. 1b).

We next examined the temporal dynamics of AS during MN differentiation. Using the RNA-seq pipeline VAST-TOOLS^21^, we identified 1599 and 1507 AS events over time in control and *VCP*^*mu*^ samples, respectively (Supplementary Fig. 2c,d). Consistent with previous studies, >60% of AS events at later stages of MN terminal differentiation were alternative exon (cassette exon inclusion and exclusion) and intron skipping events (Supplementary Fig. 2c,d)^1,6^. We found that control and *VCP*^*mu*^ samples exhibit a similar progressive increase in cassette exon inclusion over time (Fig. 1c, d) in genes enriched for cellular component organisation and axonogenesis Gene Ontology (GO) functions (Fig. 1e).

In contrast, IR accounted for >65% of AS events at the earlier phase of lineage restriction from NPC to pMN (Fig. 1c). The expression of intron-retaining transcripts increased at the transition from NPC to pMN (i.e., neural patterning) in control samples (Fig. 1f). Intriguingly, *VCP*^*mu*^ samples exhibited a striking increase in the expression of intron-retaining transcripts at the earlier developmental transition from iPSCs to NPCs (i.e. neural induction; Fig. 1f). We found that 53% of IR events started upon patterning in control samples (NPCs to pMNs); in contrast 72% of the IR events in *VCP*^*mu*^ cultures started upon neural induction (iPSCs to NPCs; Supplementary Fig. 2e, right). Importantly, 60% of all IR events involved identical introns and transcripts between the *VCP*^*mu*^ and control samples (Supplementary Fig. 2e, upper left)—these largely involve genes related to RNA processing and splicing (Fig. 1g). The remaining events were either unique to *VCP*^*mu*^ cultures or occurred at a different timepoint. This indicates that *VCP*^*mu*^ samples exhibit similar IR activity as seen in control counterparts during differentiation, but at a premature stage.

Next, we manually curated every automatically identified IR event to shortlist 167 with high-confidence. We assigned a retention value for each IR event relative to the expression of flanking exons (see Methods). It is immediately apparent from Fig. 1h that the 167 introns are systematically more highly retained in *VCP*^*mu*^ at the NPC stage compared with control samples of any stage.

As initial validation, we assessed whether the 167 introns are also retained in two independent transcriptomic data sets from human embryonic stem cells^22,23^. Examining their overall level of retention confirmed that IR during early neurogenesis is a generalisable phenomenon across diverse experimental models (Fig. 1i and Supplementary Fig. 2f). We next examined whether retained introns exhibited characteristic structural features that may discriminate them from conventionally spliced introns and found that the highly confident set of 167 retained introns are longer and more conserved than non-retained introns from same gene set (Supplementary Fig. 2g).

VCP plays a role in cell-cycle progression and an increased percentage of IR may result from the established link between splicing efficiency and the cell cycle^24^. We, therefore, examined whether the apparent acceleration in AS programmes in *VCP*^*mu*^ is explained by a mutation-dependent increase in cell-cycle activity. We employed flow cytometry on iPSCs, NPCs and pMNs treated with the fluorescent intercalating and stoichiometric agent propidium iodide to examine DNA content. These experiments effectively excluded differences in cell cycle between *VCP*^*mu*^ and control samples (Supplementary Fig. 2h–j). Thus collectively, these findings demonstrate that IR is the predominant splicing change that affects early stages of neural lineage restriction. Importantly, IR occurs both at increased levels and prematurely in *VCP*^*mu*^ cultures at the NPC stage, which cannot be explained by differences in cell-cycle activity.

### Aberrant IR in MNs carrying diverse ALS-causing mutations

**Fig. 2:**
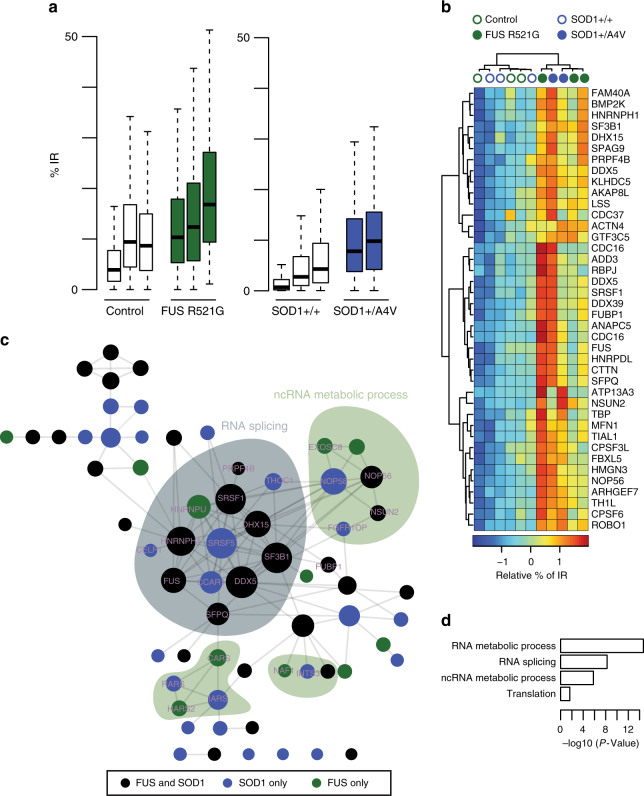
Transcripts involved in neural induction exhibit widespread intron retention in MNs derived from FUS and SOD1 ALS-causing mutations. **a** Boxplots displaying the distribution of percentage retention for 167 manually curated introns in control MNs (white box), *FUS*^*mu*^ mutant MNs (grey box) or *SOD1*^*mu*^ MNs samples (blue bar)^28,50^. Mutant samples exhibit systematically a higher proportion of IR compared with controls. Boxplots are as shown in Fig. 1h. **b** Hierarchically clustered (Manhattan distance and Ward clustering) heatmap of relative IR levels in 40 genes showing statistically significant retention during motor neurogenesis in both *SOD1*^*mu*^ and *FUS*^*mu*^ MNs. Blue circles = SOD1^*mu*^ samples, grey circles = FUS^*mu*^ samples and empty circles = control samples. **c** Network of protein–protein interactions for genes exhibiting IR during motor neurogenesis in either *SOD1*^*mu*^ or *FUS*^*mu*^ MNs. Edges represent experimentally determined protein–protein interactions annotated in the STRING database^51^. Nodes indicate proteins, coloured according to the conditions in which the corresponding transcript displays IR; circle sizes are in proportion to the number of edges in the network. **d** Bar graphs showing the enrichment scores (*P*-value from Fisher count test) of GO biological pathways associated with genes that exhibit IR during motor neurogenesis in *SOD1*^*mu*^ and/or *FUS*^*mu*^ MNs compared with controls

The pathological hallmark in >97% of all ALS cases (sporadic and familial) is nuclear-to-cytoplasmic mislocalization of TDP-43^25,26^. However, the absence of TDP-43 mislocalization from 3% of cases argue that there remain common pathogenic mechanisms yet to be discovered. To address this, we next examined the impact of SOD1 and FUS ALS-causing mutations that characteristically do not exhibit hallmark TDP-43 mislocalization. We analysed transcriptomic data sets for mutant SOD1 (*SOD1*^*mu*^*)* (*n* = 5; 2 patient-derived SOD1 A4V and 3 isogenic control FACS purified MN samples, where the mutation has been corrected)^27^ and FUS (*FUS*^*mu*^) samples (*n* = 6; 3 patient-derived FUS R521G and 3 unaffected controls MNs)^28^. We confirmed that IR is the predominant mode of splicing in MNs derived from both mutants (Supplementary Fig. 3a), suggesting a unifying molecular phenomenon across diverse genetic ALS backgrounds (VCP, SOD1 and FUS). Importantly, our high-confidence set of 167 IR events occurring prematurely during *VCP*^*mu*^ MN differentiation are also generally affected in *FUS*^*mu*^ and *SOD1*^*mu*^ MNs (Fig. 2a); specifically, 74 and 59 IR events exhibit a statistically significant increase in *SOD1*^*mu*^ and *FUS*^*mu*^ MNs, respectively, compared with controls (*P*-value < 0.01; Fisher count test; Supplementary Fig. 3b). The extent of splicing for those introns significantly retained in both *SOD1*^*mu*^ and *FUS*^*mu*^ is depicted in a heatmap in Fig. 2b. These form a network of proteins enriched in RNA splicing and translation (Fig. 2c, d). This demonstrates that the IR events identified in our ALS model also feature in MNs carrying other ALS-causing mutations that do not exhibit hallmark TDP-43 mislocalization. Cumulatively, these findings confirm the generalisability of aberrant IR across different genetic forms of ALS.

### Transient 3′ UTR remodelling in early neurogenesis

**Fig. 3:**
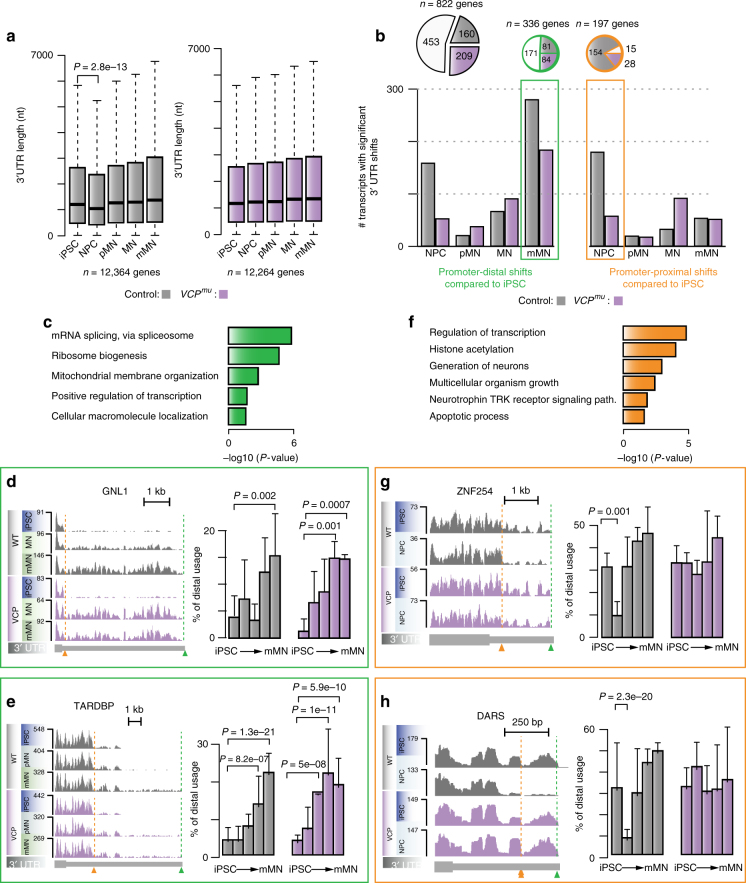
3′ UTR length variation during human motor neurogenesis. **a** Boxplots of the distributions of maximum 3′ UTR lengths expressed at distinct stages of MN differentiation in control (left) and *VCP*^*mu*^ samples (right). *P*-value obtained with Wilcoxon test. **b** Bar plots displaying the numbers of 3′ UTR with statistically significant promoter-distal (left) and promoter-proximal (right) shifts at distinct stage of differentiation compared with iPSCs in control (grey bars) and *VCP*^*mu*^ samples (magenta bars). Inset, pie charts representing the proportions of genes exhibiting alternative 3′ UTR usage in control and *VCP*^*mu*^ samples (white), control samples only (grey area) or *VCP*^*mu*^ samples only (magenta area). **c** GO enrichment analysis of biological pathways associated with genes showing statistically significant distal shifts in poly(A) site usage in control mMNs compared to control iPSCs. **d**, **e** Left, genome browser views of RNA-seq profiles in the 3′ UTRs of genes GNL1 and TARDBP which exhibit statistically significant proximal-to-distal shifts in poly(A) site usage in mMNs compared with iPSCs. Right, bar plots showing distal 3′ UTR usage relative to the proximal 3′ UTR. *P-*values obtained with Fisher count test. **f** Same as **c** for genes showing statistically significant proximal shifts in poly(A) site usage in control NPCs compared with control iPSCs. **g**, **h** Same as **d** for genes ZNF254 and DARS exhibiting statistically significant distal-to-proximal shifts in poly(A) site usage in NPCs compared with iPSCs

Splicing and polyadenylation regulation are often interconnected. To examine whether 3′ UTR length varies throughout differentiation and how the *VCP*^*mu*^ affects this process, we first extended the current catalogue of Ensembl 3′ UTR annotation using our RNA-seq data (see methods); for each gene, we then analysed their maximum lengths expressed over the course of differentiation. In both control and *VCP*^*mu*^ samples, the average 3′ UTR lengths for 12,364 continuously expressed genes increase during the differentiation time course. Exceptionally though, 3′ UTRs in the control samples are temporarily shortened during the NPC stage (*P*-value < 0.01; Wilcoxon test; Fig. 3a). As 3′ UTR lengthening is expected upon embryonic development, the observed shortening at the NPC (compared to iPSC) stage is surprising.

To investigate further the dynamics of 3′ UTR processing, we analysed tandem poly(A) sites that were located on the same terminal exon. Using the number of reads mapped to the terminal 300 nt segment of each 3′ UTR as a proxy for the isoform expression level, we quantified alternative poly(A) site use at different developmental stages compared with iPSCs. 822 genes display statistically significant 3′ UTR changes in either control and/or *VCP*^*mu*^ (Fig. 3b). In 171 genes, there is increased use of the distal poly(A) site in both control and *VCP*^*mu*^ mMNs (Fig. 3b), consistent with a previous study^29^; these genes are enriched in GO terms related to mRNA splicing (Fig. 3c). At the MN stage <10 genes display significant differential 3′ UTR usage when directly comparing control and *VCP*^*mu*^ cultures, confirming similar 3′ UTR length regulation upon terminal differentiation between control and *VCP*^*mu*^ (Supplementary Fig. 4a). It is noteworthy that in several cases, *VCP*^*mu*^ samples exhibited a significant promoter-distal shift at a comparatively earlier stage to control samples. These examples include GNL1 (Fig. 3d) and TARDBP (Fig. 3e), the latter also exhibiting accumulation of long 3′ UTR isoform in *SOD1*^*mu*^ MN compared to controls, but not in *FUS*^*mu*^ (Supplementary Fig. 4c,d). None of these 3′ UTR variation led to measurable changes in gene or protein expression (Supplementary Fig. 4e,f,g).

Of the 822 genes, 169 exhibit statistically significant promoter-proximal shift specifically in control NPCs compared with iPSCs (Fig. 3b). Various biological pathways such as transcription and neurotrophin regulation are represented among the transcripts exhibiting transient 3′ UTR shortening (Fig. 3f). Approximately 80% of these events are absent in *VCP*^*mu*^ (Fig. 3b,g,h); this is further confirmed when *VCP*^*mu*^ and control samples are directly compared at the NPC stage (Supplementary Fig. 4a,b). These data demonstrate that extensive 3′ UTR length variation peaks prior to IR and characterises the transition from iPSCs to NPCs. Importantly, *VCP*^*mu*^ samples do not exhibit any apparent similar process.

### Splicing factor downregulation coincides with aberrant IR

**Fig. 4:**
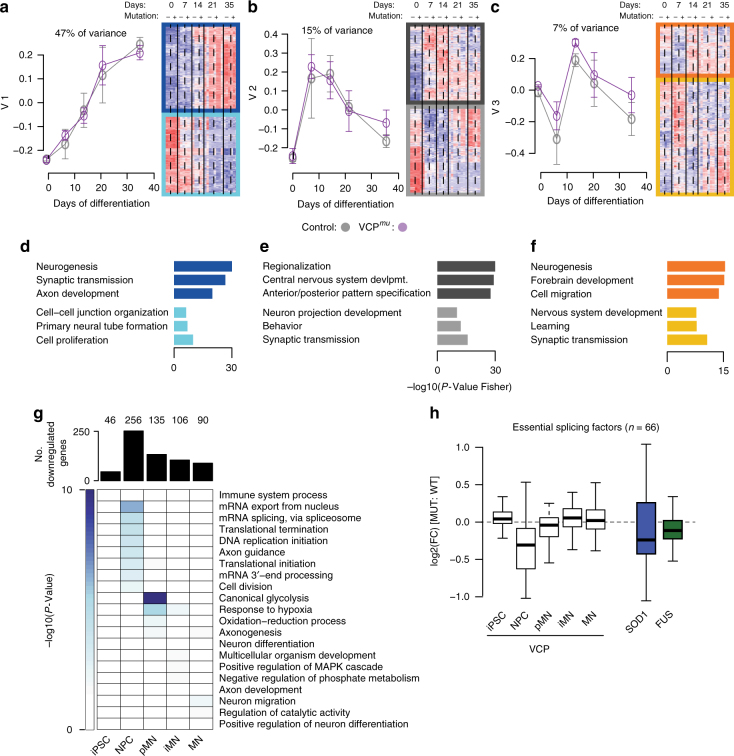
Global downregulation of splicing components coincides with IR. **a**–**c** Singular value decomposition analysis of the expression of 15,989 genes in *n* = 31 samples. Left, Line plots showing the expression profiles of the first three singular vectors $\overrightarrow v _1$ through $\overrightarrow v _3$, capturing 47%, 15% and 7% of the variance in gene expression, respectively. Grey and magenta data points indicate expression for the control and *VCP*^*mu*^ samples. Right, heatmap of the standardised expression of genes whose expression profiles correlate positively (indicated by top three darker rectangles) and negatively (indicated by bottom three lighter rectangles) with the first three right singular vectors. **d**–**f** Bar plots displaying the enrichment scores for GO biological functions of genes that correlate positively (top three bars) or negatively (bottom three bars) with the first three right singular vectors. **g** Upper, bar plots depicting the numbers of downregulated genes in *VCP*^*mu*^ samples compared with control at distinct stages of MN differentiation. Lower, heatmap of the GO biological functions enriched among downregulated genes at the corresponding time-points. **h** Boxplots showing the distributions of log2 fold-changes for 66 essential splicing factor genes between ALS mutants and controls; white boxes = *VCP*^*mu*^ compared with controls, blue box = *SOD1*^*mu*^ compared with isogenic controls, green box = *FUS*^*mu*^ compared with controls

Having identified major splicing differences in ALS samples compared with control, we next sought to understand whether the ALS genetic background drives measurable transcriptional differences during motor neurogenesis. Specifically, we investigated expression changes that are not optimally identified by hierarchical clustering (Fig. 1b), which is often dominated by a single transcriptional programme. We applied singular value decomposition (SVD) to the entire expression matrix (15,989 genes across 31 samples; Fig. 1a) to identify the major transcriptional programmes and associated biological processes underlying motor neurogenesis^30^. Using this method we find that (1) progressive neurogenesis, (2) transient neural induction and patterning, and (3) terminal differentiation with some contribution to neural induction compose the main orthogonal cellular expression programmes that operate along the developmental time course; these first three components capture 69% of the variance in gene expression (Fig. 4a–c, Supplementary Fig. 5a). GO functional enrichment analysis of the groups of genes that were either positively or negatively associated with these components (Fig. 4d–f) further confirms the biological association of each major component. Importantly, control and *VCP*^*mu*^ samples behave similarly in these first three components. A multivariate linear analysis confirmed that time in culture rather than VCP mutation is the variable explaining components 1, 2 and 3 (Supplementary Fig. 5b).

We next conducted differential gene expression analysis to assess whether specific genes are differentially regulated at different stages of neuronal development in *VCP*^*mu*^ compared with control samples. Two hundred and fifty-six genes were statistically significantly downregulated in *VCP*^*mu*^ at the NPC stage during which aberrant IR occurs (log2FC > 1.5 and *P*-value < 0.01); these genes are highly enriched in mRNA splicing and 3′ end processing GO functions (Fig. 4g). It is noteworthy that 47 of these genes encode RBPs (Supplementary Fig. 5d). There were far fewer upregulated genes (Supplementary Fig. 5c). We also examined expression levels of 66 known splicing regulator genes^31^; there is clear global downregulation at the NPC stage of *VCP*^*mu*^ samples and in independent *SOD1*^*mu*^ and *FUS*^*mu*^ MNs coinciding with aberrant IR in each case (Fig. 4h).

In summary, we find that transcriptional and RNA processing programmes reflect the developmental trajectory of MNs as specific pathways are activated in a stage-directed manner. Despite post-transcriptional defects, the primary transcriptional programme is not disrupted during MN differentiation by the VCP mutation. However, a global downregulation in expression of splicing components does occur concomitantly with aberrant IR upon diverse ALS-causing mutations.

### Cytoplasmic IR transcripts and nuclear loss of SFPQ protein

**Fig. 5:**
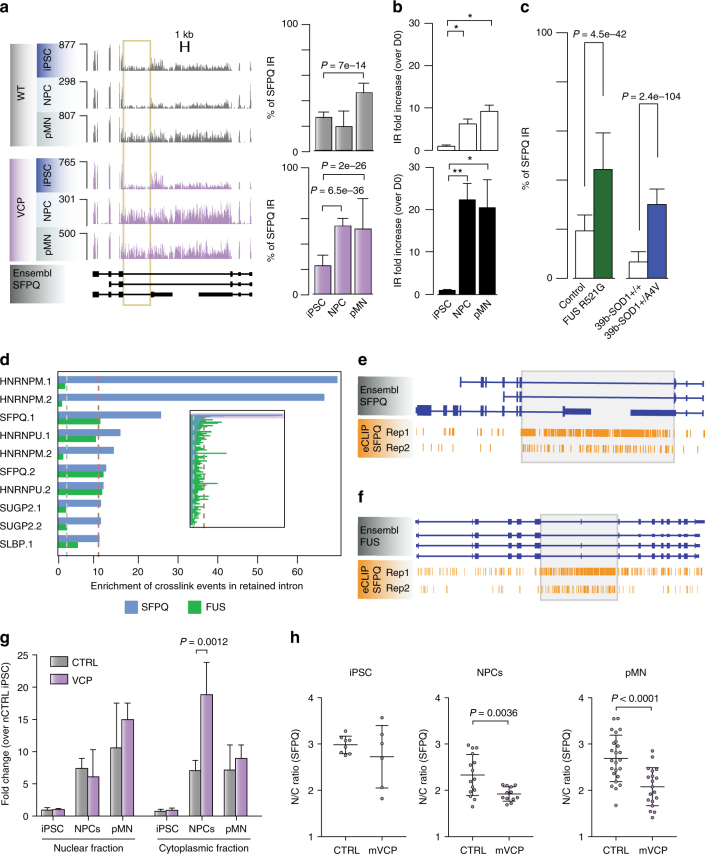
Aberrant SFPQ intron retention in diverse genetic forms of ALS and its interplay with the SFPQ protein. **a** Left, genome browser views of RNA-seq profiles for the intron-retaining gene SFPQ in control and *VCP*^*mu*^ samples at iPSC, NPC and pMN stages. 9 kb intron 9/9 of interest is indicated with yellow box. Right, bar graphs quantifying percentage IR across the entire time course in control and *VCP*^*mu*^ samples (mean ± s.d.; Fisher count test). **b** Bar plots displaying SFPQ IR levels measured by qPCR; white bars = controls, black bars = *VCP*^*mu*^. IR levels at each timepoint were compared with that of the iPSC stage of the same group. *N* = 3 control lines and *N* = 4 VCP lines (mean + s.d. *p < 0.05, **p < 0.01, one-way ANOVA with Dunnet correction for multiple comparisons). **c** Bar plots showing the percentage SFPQ IR for FUS^*mu*^ or SOD1^*mu*^ MNs in control compared with (mean ± s.d.; Fisher count test). **d** Bar plots depicting the level of enrichment in RBP-binding to the retained intron compared with the non-retained introns within the same gene; blue bars = SFPQ gene, green bars = FUS gene. **e**–**f** Genome browser view of SFPQ eCLIP crosslinking events along SFPQ and FUS transcript annotations. Grey boxes highlight the location of retained introns. **g** Bar plots showing the level of nuclear and cytoplasmic localisation of IR transcripts measured by qPCR. IR levels in each fraction were compared with that of the nuclear fraction of control iPSCs. *N* = 3 control lines and *N* = 4 VCP lines, mean + s.d. *P*-value from two-way ANOVA with Tukey correction for multiple comparisons. **h** Subcellular localisation of SPFQ determined by immunocytochemistry in iPSCs, NPCs and pMNs. The ratio of the average intensity of the SFPQ staining in the nucleus (N) vs cytoplasm (C) was automatically determined in both CTRL and *VCP*^*mu*^ cells. Data shown is average N/C ratio (±s.d.) per field of view from four control and four *VCP*^*mu*^ lines. *P*-value from unpaired *t*-test with Welch’s correction. See also Supplementary Fig. 7d

The Drosophila behaviour human splicing (DBHS) family member *SFPQ* gene encodes a protein that plays key roles in transcription, splicing, 3′ end processing and axon viability^32–35^. Here, the 9 kb intron 9/9 in the SFPQ gene displays the most prominent IR event identified during motor neurogenesis in our *VCP*^*mu*^ NPC samples compared with control counterparts. Importantly, aberrant SFPQ IR also occurs in *SOD1*^*mu*^ and *FUS*^*mu*^ MNs compared with controls (Fig. 5a, c). We validated SFPQ IR and its deregulation in *VCP*^*mu*^ compared to control samples by qPCR analysis from multiple independent iPSC lines (three clones from three healthy controls and four clones from two patients with VCP mutations; Fig. 5b and Supplementary Fig. 6a). We further validated two splicing regulators FUS and DDX39, that are similarly strongly affected in *VCP*^*mu*^ NPCs, and in *SOD1*^*mu*^ and *FUS*^*mu*^ MN (Supplementary Fig. 6b–g).

We examined large-scale ENCODE eCLIP data for 112 RBPs in K562 and HepG2 cells to identify candidates that bind the retained introns in SFPQ and FUS^36,37^. Ranking these RBPs by the proportion of crosslinking events in the retained intron compared with non-retained introns in the same gene revealed SFPQ and HNRNPM as the most highly enriched RBPs (Fig. 5d–f and Supplementary Fig. 7a). This indicates that the SFPQ protein directly binds to the SFPQ and FUS retained introns.

Having uncovered evidence of interactions between the SFPQ protein and SFPQ intron 9/9, we sought to understand the nature of this crosstalk. Changes in IR are not accompanied by a statistically significant difference in either gene or protein expression levels between *VCP*^*mu*^ and control samples (Supplementary Fig. 7b,c). Instead we used nuclear-cytoplasmic fractionation and identify statistically increased abundance of the SFPQ intron-retaining isoform in the *VCP*^*mu*^ cytoplasm compared with control cultures (Fig. 5g). These findings together prompted investigation of SFPQ protein subcellular distribution. We found a modest but statistically significant nuclear loss of SFPQ protein in *VCP*^*mu*^ cultures (Fig. 5h).

In summary, the most significant increase of IR across VCP, FUS and SOD1 is seen in the SFPQ transcript. This intronic sequence is bound extensively by the SFPQ protein and both members of this complex (i.e. the protein and the intron-retaining transcript) exhibit aberrant cellular distribution.

### Nuclear loss of SFPQ across familial and sporadic ALS

**Fig. 6:**
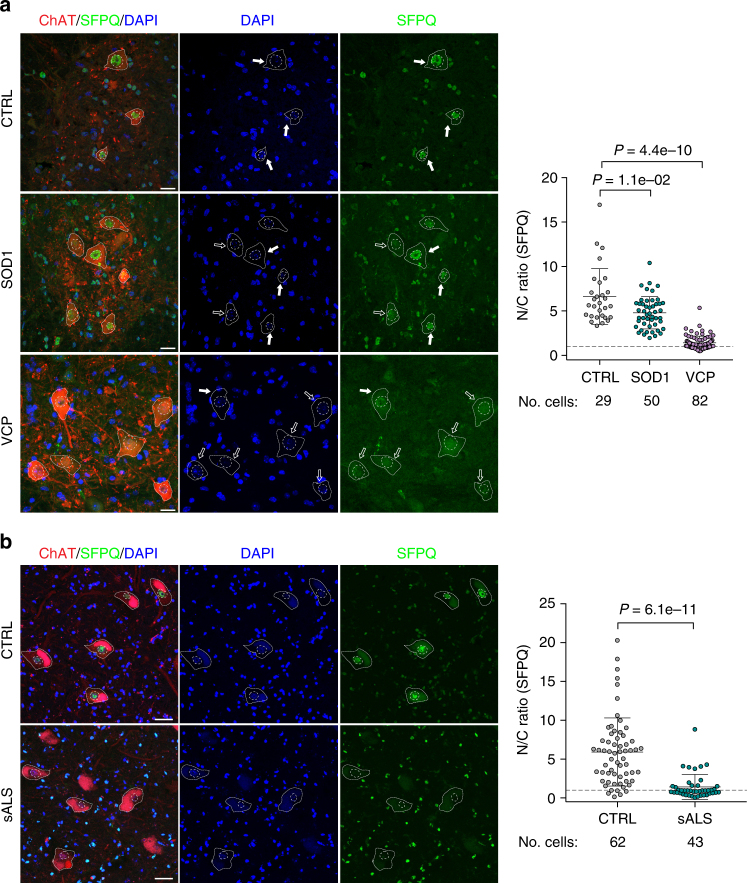
SFPQ nuclear clearance is a molecular hallmark of genetic and sporadic ALS. **a** Analysis of the subcellular localisation of SFPQ in MNs in the ventral spinal cord of wild-type, *VCP*^*A232E*^ and *SOD1*^*G93A*^ mice. MN cytoplasm was identified by ChAT staining, nuclei were counterstained with DAPI. Data shown is nuclear/cytoplasmic (N/C) ratio (mean ± s.d.) per cell from three wild-type, 4 *SOD1*^*G93A*^ and 3 *VCP*^*A232E*^ mice. Scale bar: 20 μm. *P*-values from one-sided Welch’s *t*-test. Cells from individual animals in each condition were pooled together after excluding individual mice effect by comparing full linear (disease and individual factor) with reduced linear (disease only) models using the Akaike Information Criterion. **b** Analysis of the subcellular localisation of SFPQ in MNs in the ventral spinal cord of healthy controls and patients with sporadic ALS (sALS). MN cytoplasm was identified by ChAT staining, nuclei were counterstained with DAPI. Only MNs with a visible nucleus were considered for the analysis. Scale bar 50 μm. Data shown is N/C ratio (mean ± s.d.) per cell from three cases per group

Having established decreased nuclear abundance of the SFPQ protein in differentiating iPSC cultures, we sought to test the generalisability of this finding across in vivo mouse transgenic ALS models and human post-mortem tissue from sporadic ALS cases. To this end, we first examined two mouse transgenic models of ALS including *SOD1*^*G93A*^ and *VCP*^*A232E*^. Using this approach, we demonstrate nuclear loss of the SFPQ protein from spinal cord MNs with both ALS-related gene mutations (Fig. 6a). Nuclear loss of the protein thus arises in different genetic forms of ALS known to exhibit TDP43 proteinopathy (*VCP*^*mu*^) and those that do not (*SOD1*^*mu*^). Noting that 90% of ALS cases are non-familial, we next examined spinal cord post-mortem tissue from sporadic human ALS cases. Indeed, we demonstrate striking nuclear loss of SFPQ protein in human sporadic ALS compared with normal control tissues, underscoring the wider relevance of this finding (Fig. 6b). From these results we conclude that nuclear loss of SFPQ protein is a unifying hallmark across diverse in vitro and in vivo models representing familial and sporadic ALS.

## Discussion

The objectives of this study were twofold: (i) to resolve the alternative RNA processing events underlying distinct stages of MN lineage restriction and (ii) to search for common primary pathogenic mechanisms among pathologically divergent forms of ALS (i.e. with or without TDP43 proteinopathy). In order to achieve this, we integrated the directed differentiation of patient-specific iPSCs into spinal MNs with RNA sequencing and comprehensive bioinformatic examination. We showed that a robust splicing programme underlies MN development as summarised in Fig. 6a. In resolving the precise nature of sequential post-transcriptional programmes underlying distinct stages of MN differentiation, we revealed that the timing of these carefully choreographed molecular events is perturbed by the ALS-causing VCP mutation. In analysing additional ALS-related MNs samples, we further showed that ALS gene backgrounds, such as SOD1 and FUS, affect the transcript structure of key RNA splicing regulators in a similar manner to those targeted by the aberrant splicing programme in VCP samples. We further show that a global downregulation of splicing components coincides with the splicing defects. This “weakening” of the splicing machinery provides a plausible explanation for the aberrant IR we found in VCP, SOD1 and FUS mutation-related ALS. The SFPQ transcript exhibits the most significantly retained intron, to which the SFPQ protein binds avidly, providing evidence of a direct interaction between the protein and intron-retaining transcript. We also demonstrated that the SFPQ intron-retaining transcript is exported to the cytoplasm. This occurs to a greater degree in the VCP mutant coinciding with the aberrant IR programme. This prompted us to examine the cellular localisation of the SFPQ protein, which uncovered its nuclear loss in ALS. SFPQ encodes a key regulator of axonal mRNA localisation and axonal development^38^. Taken together, these results raise the idea that dysregulated transcript structure may play a key role in axonal degeneration during ALS development. Indeed SFPQ is emerging as a key regulator of neurodegeneration more widely^34,38–40^.

**Fig. 7:**
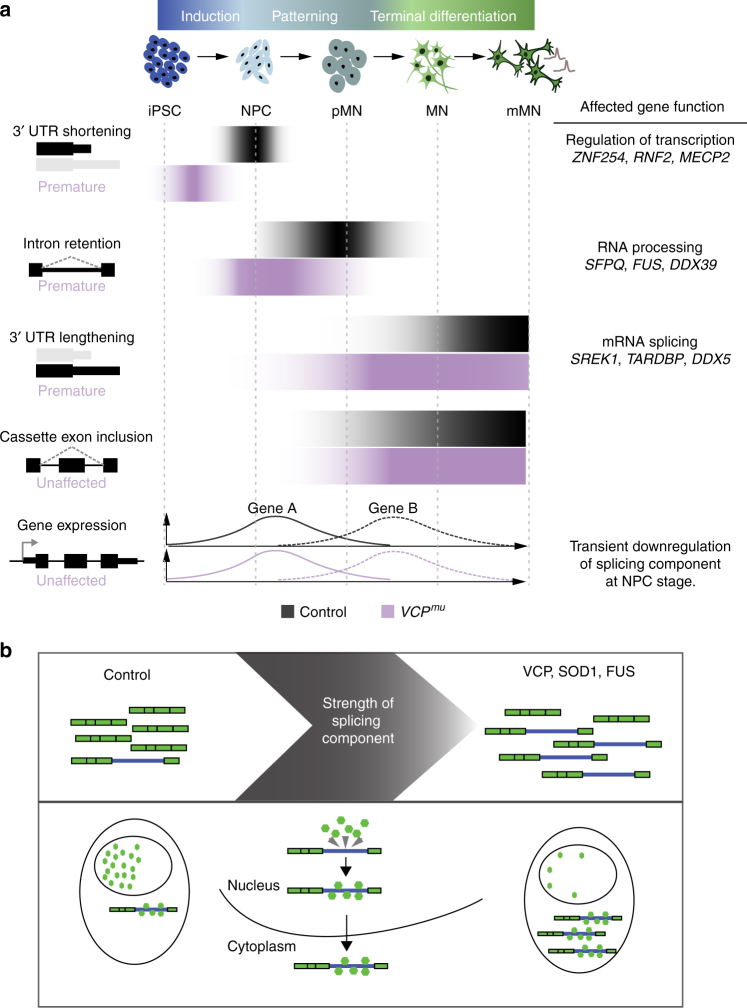
Schematic diagram of proposed model. **a** Cartoon summarising the time course of post-transcriptional events underlying human motor neurogenesis in control and *VCP*^*mu*^ samples. Our results suggest that the predominant RNA-processing events at an early stage of neural differentiation are IR and 3′ UTR remodelling. These events start prematurely in *VCP*^*mu*^ samples but do not affect major transcriptional programmes underlying human MN differentiation. **b** Cartoon summarising the functional consequence of aberrant IR in SFPQ gene across diverse genetic and sporadic forms of ALS. The 9 kb intron 9/9 in SFPQ is retained across all ALS-mutant backgrounds, which leads to increased cytoplasmic abundance of affected transcripts. The SFPQ protein binds extensively to the retained intron. The SFPQ protein itself is relocalised from the nucleus to the cytoplasm in all ALS-mutant backgrounds, as well as post-mortem samples of sporadic ALS patients

Dynamic AS changes have been previously reported in rodent forebrain between different developmental stages^41,42^. Here we have identified previously unrecognised modifications in the transcript structure of genes regulating RNA processing and splicing at early stages of human MN lineage restriction (Fig. 7a). We showed that a peak of 3′ UTR remodelling, including 3′ UTR shortening, precedes IR. Specifically there is statistically significant 3′ UTR length variation affecting transcript structures as cells exit pluripotency during neural induction. This precedes the peak of intron-retaining transcript accumulation that occurs upon neural patterning to the ventral spinal cord. Following this, progressive 3′ UTR lengthening, cassette exon inclusion and intron splicing then characterise the remaining phases of terminal differentiation to MNs as previously shown^1,6^.

3′ UTRs play key roles in post-transcriptional gene expression regulation and mRNAs expressed in brain tissues generally have longer 3′ UTRs compared to other tissues^12,13^. Indeed progressive lengthening of mRNA 3′ UTRs is observed during mouse embryonic development^29^. Here we observed extensive 3′ UTR length variation during neural induction from human iPSCs. Unexpectedly, we found a transient shortening of 3′ UTRs preceding the previously reported progressive 3′ UTR lengthening during terminal differentiation. The molecular function of such shortening remains unclear, although other studies report a potential role of the 3′ UTR in paraspeckle formation and the exit of pluripotency^43,44^. The highly transient nature of this regulatory process may explain why it has evaded experimental detection to date. Our time-resolved experimental design has permitted the identification of sequential transcriptional programmes underlying human motor neurogenesis.

IR is emerging as novel post-transcriptional mechanism regulating gene expression. Neural and immune cell types have higher proportions of retained introns compared to other tissues^7^. Previous studies have demonstrated progressive IR during terminal differentiation of mouse neurons and the relevance of intron-retaining transcripts in regulating the expression of functionally linked neuron-specific genes in mouse models^6,7^. Here we provided evidence for a transient increase in intron-retaining transcripts at the pMN stage that targets a network of splicing regulators. Recognising the interaction between IR events and nuclear paraspeckles, this raises the possibility that either these transcripts contribute to the stabilisation of the paraspeckles or that they are dynamically sequestered in such nuclear structures in order to inhibit their function. Future experiments will determine the stability and localisation of such transcripts.

We showed that the timing of post-transcriptional programmes is perturbed in samples with ALS-causing VCP mutations, with premature and increased IR and to some extent 3′ UTR length variation. Interestingly, key RNA splicing regulators targeted by the aberrant splicing programme in *VCP*^*mu*^ samples also exhibited widespread IR in MNs harbouring mutations in other ALS-causing genes, including SOD1 and FUS. Notably, we observed reduced expression of key components of the spliceosome coinciding with aberrant IR in VCP, SOD1 and FUS. This is consistent with a recent study showing association between IR and reduced recruitment of splicing factors^45^. Although the aberrant splicing programme in *VCP*^*mu*^ samples might be linked to neuronal degeneration, the lack of changes in transcriptional programmes associated with motor neurogenesis indicate that the dysfunctions in IR and 3′ end processing do not fundamentally affect neuronal development. These findings together raise the hypothesis that aberrant IR represents subtle manifestations of a dysregulated pathway of the developing neurons, which eventually contributes to the increased vulnerability of mature MNs.

The most significant intron-retaining transcript across *VCP*^*mu*^, *SOD1*^*mu*^ and *FUS*^*mu*^ iPSC models was SFPQ, which encodes a protein that plays key roles in salient ALS-related pathways including RNA transcription, splicing, 3′ end processing and axon viability^32–34^. Subsequent experiments showed that the intron-retaining transcript is more abundant in the cytoplasm of *VCP*^*mu*^ compared to control counterparts, and coincides with nuclear decrease of the SFPQ protein. We also found that the SFPQ protein binds extensively to its retained intron. Autoregulation among RNA-binding proteins (RBPs) (e.g. TDP43) is recognized and so this finding is consistent with previous literature^46,47^. Crucially, we find nuclear loss of SFPQ protein in both mouse transgenic models of SOD1 and VCP, and in human post-mortem sporadic ALS spinal cord, suggesting that this represents a molecular hallmark of ALS. We therefore propose that the interaction between the SFPQ protein and its intron-retaining transcript imposes their aberrant co-localisation in the cytoplasm (Fig. 7b). The reduced abundance of nuclear SFPQ protein may impair pre-mRNA splicing, which might contribute to the post-transcriptional defects we and others have identified in ALS^48,49^. Taken together, we find that IR in the SFPQ transcript and nuclear loss of the SFPQ protein are common molecular hallmarks across diverse genetic and sporadic forms of ALS.

## Methods

### Ethics statement

Informed consent was obtained from all patients and healthy controls in this study. Experimental protocols were all carried out according to approved regulations and guidelines by UCLH’s National Hospital for Neurology and Neurosurgery and UCL’s Institute of Neurology joint research ethics committee (09/0272).

### Derivation of human fibroblasts and iPSC

Dermal fibroblasts were cultured in OptiMEM + 10% FCS medium. The following episomal plasmids were transfected for iPSC generation: pCXLE hOct4 shp53, pCXLE hSK and pCXLE hUL (Addgene), as previously reported^56^. Details of the lines used in this study are provided in Supplementary Table 1. Two of the control lines used (control 2 and control 3) are commercially available and were purchased from Coriell (cat. number ND41866*C) and ThermoFisher Scientific (cat. number A18945), respectively.

### Cell culture

Induced PSCs were maintained on Geltrex (Life Technologies) with Essential 8 Medium media (Life Technologies), and passaged using EDTA (Life Technologies, 0.5 mM). All cell cultures were maintained at 37 C and 5% carbon dioxide.

### Motor neuron differentiation

MN differentiation was carried out using an adapted version of a previously published protocol^19^. Briefly, iPSCs were first differentiated to neuroepithelium by plating to 100% confluency in chemically defined medium consisting of DMEM/F12 Glutamax, Neurobasal, L-Glutamine, N2 supplement, non-essential amino acids, B27 supplement, β-mercaptoethanol (all from Life Technologies) and insulin (Sigma). Treatment with small molecules from day 0–7 was as follows: 1 µM Dorsomorphin (Millipore), 2 µM SB431542 (Tocris Bioscience), and 3.3 µM CHIR99021 (Miltenyi Biotec). At day 8, the neuroepithelial layer was enzymatically dissociated using dispase (GIBCO, 1 mg ml^−1^), plated onto laminin coated plates and next patterned for 7 days with 0.5 µM retinoic acid and 1 µM Purmorphamine. At day 14 spinal cord MN precursors were treated with 0.1 µM Purmorphamine for a further 4 days before being terminally differentiated for >10 days in 0.1 µM Compound E (Enzo Life Sciences) to promote cell-cycle exit. At relevant timepoints, cells were harvested for RNA extraction or fixed in 4% paraformaldehyde for immunolabelling.

### RNA extraction and sequencing

The Promega Maxwell RSC simplyRNA cells kit including DNase treatment, alongside the Maxwell RSC instrument, was used for RNA extractions. For qPCR validations, RNA was extracted using the RNeasy Plus Mini Kit (Qiagen). The nanodrop was used to assess RNA concentration and the 260/280 ratio, and the Agilent bioanalyser was used to assess quality. RNA integrity (RIN) scores were >8 for all samples used in this work. RNAseq libraries were prepared using the Truseq stranded mRNA kit (Illumina) with 1 µg input poly(A) + RNA. The products were then purified and enriched with PCR amplification to create the final cDNA libraries. Libraries were sent for high-throughput sequencing, run for 75 cycles on a rapid flow cell, at the Institute of Neurology’s NGS core facility using the Hiseq2500. For the analysis of nuclear and cytoplasmic transcripts the Cytoplasmic and Nuclear RNA Purification Kit (Norgen Biotek Corp) was used.

### RNA-sequencing data

Single-end stranded RNA-seq reads of 50 bp were obtained from five distinct stages of MN differentiation from control and *VCP*^*mu*^ samples (iPSC, and days 7, 14, 21 and 35); samples are listed in Supplementary Data 1. The Gene Expression Omnibus (GEO) accession number for the RNA-seq libraries is GSE98290. We also obtained single- and paired-end RNA sequencing reads derived from two independent studies of in vitro neural differentiation of hESCs (GSE20301^22^ and GSE86985^23^); two independent studies on familial forms of ALS either caused by mutant SOD1 (*n* = 5; 2 patient-derived SOD1A4V and 3 isogenic control MN samples where the mutation has been corrected; Hb9 FACS purified MNs, GSE54409^27^ or FUS (*n* = 6; 3 patient-derived FUS R521G and 3 healthy sibling controls MNs, GSE77702^28^; two RNA-seq data from human ESC shared by Miha Modic; Two foetal spMN (E-MTAB-3871; NIH Roadmap Epigenomics Mapping Consortium) and adult spMN (laser-captured spMN; GSE76514^53^) samples.

### Expression data pre-processing

Single- and paired-reads for each of the study were initially aligned to ribosomal RNA sequences to filter out reads that may come from ribosomal RNA contamination using bowtie2 (-v 0)^57^. The remaining reads were aligned to the human genome (h19) using the splice aware aligner TopHat2^58^ with default parameters. All libraries generated in this study had <1% rRNA, <1% mtDNA, >90% strandedness and >70% exonic reads.

### Gene quantification and unsupervised characterisation of the data-set

The absolute quantification of the genes was performed using HTSeq count^59^. Subsequent analysis was performed with the R statistical package version 3.3.1 (2016) and Bioconductor libraries version 3.3 (R Core Team. R: A Language and Environment for Statistical Computing. Vienna, Austria: R Foundation for Statistical Computing; 2013).

Prior to unsupervised clustering analysis of the 31 samples of MN differentiation, we identified reliably expressed genes for each condition (*VCP*^*mu*^ or control at days 0, 7, 14, 21 and 35). For a given sample, the histogram of log2 gene count is generally bimodal, with the modes corresponding to non-expressed and expressed genes. Reliably expressed genes were identified by fitting a two-component Gaussian mixture to the log2 estimated count gene data with R package mclust^60^. A gene was considered to be reliably expressed in a given condition if the probability of it belonging to the non-expressed class was under 1% in each sample belonging to the condition. 15,989 genes were selected based on their detected expression in at least one of the 10 conditions (i.e. five different timepoints of lineage restriction for control and *VCP*^*mu*^). Next we quantile normalised the columns of the gene count matrix with R package limma^61^. Unsupervised hierarchical clustering of the filtered and normalised gene count matrix was performed with Spearman rank correlation as a distance measure and complete clustering algorithm.

We performed SVD of the expression of the 15,989 genes across the five distinct stages of MN differentiation from healthy controls and *VCP*^*mu*^. We then selected the components maximally capturing variance in gene expression. To visualise the right singular vectors $\left\{ {\overrightarrow v _k} \right\}$, we plotted the expression on the vertical axis as a function of the time corresponding to each sample on the horizontal axis and colouring all samples corresponding to healthy controls grey, and those corresponding to *VCPmu* in magenta. Next we identified genes whose expression profiles correlated (Pearson correlation between individual gene expression profile and right singular vectors) and contributed (projection of each individual gene expression profile onto right singular vectors) most strongly (either positively or negatively) with the expression profile of the singular vectors. In order to identify representative genes for each singular vector, the genes were ranked according to both projection and correlation scores. The highest (most positive scores in both projection and correlation) and lowest (most negative scores in both correlation and projection) genes were selected for each singular vector using K-mean clustering for downstream GO enrichment analysis.

### Analysis of APA from RNA-seq

For the identification of alternative 3′ UTR identification from RNA-seq, nucleotide-level stranded coverage was obtained for each of the 31 samples using genomecov from the BEDTools suite^62^. Next continuously transcribed regions were identified using a sliding window across the genome requiring a minimum coverage of seven reads in more than 80 positions per window of 100 bp; neighbouring regions separated by low-mappable regions were merged as previously described^13^. Expressed fragments were then associated with overlapping 3′ UTR using the latest hg19 Ensembl versions v75^63^. Isolated expressed regions that did not overlap with any feature were further associated with the closest 3′ UTR if (1) the closest annotated feature was nothing but a 3′ UTR, (2) if the strand of the expressed region was in line with the strand of the closest 3′ UTR, and (3) if the distance to the 3′ UTR was less than 10’000 kb, which is the range of intragenic distance. The resulting extended 3′ UTRs were subjected to extensive filtering to exclude potential intragenic transcription, overlapping transcripts and retained introns as previously described^13^. We finally intersected the longest 3′ UTR segment annotated with RNA-seq data with a poly(A) site annotation built using reads from 3′-end sequencing libraries in human samples^64^ to obtain putative poly(A) within longest 3′ UTR segment of each transcript.

We then used the number of reads mapped to −300 nt terminal region of each 3′ UTR isoforms as a proxy for the 3′ UTR isoform expression level to identify changes in the use of proximal and distal poly(A) sites. The density of mapped reads in −300 nt terminal region of 3′ UTR isoform is bimodal, with a low-density peak probably corresponding to background transcription i.e. 3′ UTR isoforms of low abundance or 3′ UTR isoforms to which reads were spuriously mapped, and a high-density peak corresponding to expressed 3′ UTR isoforms. In order to identify reliably expressed 3′ UTR isoforms in the study, a two-component Gaussian mixture was fitted to the data using the R package mclust^60^; an isoform was called reliably expressed if in both replicates had less than 1% chance of belonging to the background category.

In order to identify transcripts that show a marked change in the poly(A) site usage between conditions, we scored the differences in proximal-to-distal poly(A) site usage using the following two scores:

$$S_1 = {\mathrm{log}}_2\left( {\frac{{I_{\mathrm{proximal}}}}{{I_{\mathrm{distal}}}}} \right)_{\mathrm{Cond1}} - {\mathrm{log}}_2\left( {\frac{{I_{\mathrm{proximal}}}}{{I_{\mathrm{distal}}}}} \right)_{\mathrm{Cond2}}$$

$$S_2 = \frac{{I_{\mathrm{proximal}}}}{{I_{\mathrm{proximal}} + I_{\mathrm{distal}}}}_{\mathrm{Cond1}} - \frac{{I_{\mathrm{proximal}}}}{{I_{\mathrm{proximal}} + I_{\mathrm{distal}}}}_{\mathrm{Cond2}} \in \left[ { - 1,1} \right]$$

The statistical significance of the changes in proximal-to-distal poly(A) site ratio between two conditions was assessed by Fisher’s exact count test using summed-up raw read counts of promoter-proximal versus promoter-distal 3′ UTR isoforms originating either conditions. We adjusted the *P*-value controlling for False Discovery Rate (FDR) of 0.01. We restricted our analysis on Ensembl transcripts containing at least two 3′ UTRs generated by tandem polyadenylation expressed in conditions of interest. Proximal shifts were then selected when $S_1 \le - 1$, $S_2 \le - 15\%$ and FDR < 0.01; distal shift were selected when selected when $S_1 \ge 1$, $S_2 \ge 15\%$ and FDR < 0.01.

### Splicing analysis

The identification of all classes of AS events in MN differentiation was performed with the RNA-seq pipeline vast-tools^21^. For an AS event to be considered differentially regulated between two conditions, we required a minimum average ΔPSI (between the paired replicates) of at least 10% and that the transcript targeted by the splicing event in question to be reliably expressed in all samples from the conditions compared i.e. enough read coverage in all samples of interest. We next conducted Integrative Genomics Viewer (IGV)-guided manual curation to remove low coverage IR obtaining 167 high-confidence IR events. IR focused analysis has next been performed on these 167 IR events for which a percentage of IR has been calculated as the fraction of intron mapping reads to the average number of reads mapping to the adjacent 5′ and 3′ exons normalised to the length of the respective intron and exons. A Fisher count test *P*-value has been obtained when testing for differential IR between conditions.

### Differential gene expression analysis

For differential gene expression analysis we ran Kallisto^54^ and Sleuth^55^. Kallisto was used to (1) build a transcript index from the Ensembl GRC38 release 85 Homo sapiens transcriptome (-k 31), (2) pseudo-align the RNA-seq reads to the transcriptome and (3) quantify transcript abundances (-b 100—single -l 275 -s 50—rf-stranded). We next identified differentially expressed genes with Sleuth. Subsequent analysis was performed with the R statistical package version 3.3.1 (2016) and Bioconductor libraries version 3.3 (R Core Team. R: A Language and Environment for Statistical Computing. Vienna, Austria: R Foundation for Statistical Computing; 2013). Prior to selecting differentially expressed genes we identified reliably expressed genes. Kallisto outputs transcript abundance, and thus we calculated the abundance of genes by summing up the estimated raw count of the constituent isoforms to obtain a single value per gene. For a given sample, the histogram of log2 gene/transcript count is generally bimodal, with the modes corresponding to non-expressed and expressed genes. Reliably expressed genes/transcripts were next identified by fitting a two-component Gaussian mixture to the log2 estimated count transcript and gene data R with package mclust^60^; a pseudocount of 1 was added before log2 transformation. A gene was considered to be reliably expressed in a given condition if the probability of it belonging to the expressed class was above 0.1 in each sample belonging to the condition. Finally, genes that showed a log twofold differential expression and a *P*-value < 0.05, and that were reliably expressed in either VCP mutant or control condition were considered as changing significantly.

### GO enrichment analysis

GO enrichment analysis was performed using classic Fisher test with topGO Bioconductor package^65^. Only GO terms containing at least 10 annotated genes were considered. A *P*-value of 0.05 was used as the level of significance. On the figures, top significant GO terms were manually selected by removing redundant GO terms and terms which contain fewer than five significant genes.

### Network analysis

Experimental protein–protein interaction information has been retrieved from the STRING database^66^ and visualised in Cytoscape^67,68^.

### Mapping of eCLIP data

Raw eCLIP data were downloaded from ENCODE^36,37^. Before alignment, two-stage adaptor removal was performed using Cutadapt according to the ENCODE eCLIP standard operating procedure. A two-stage approach was also used for alignment. First, Bowtie2 was used to remove reads aligning to rRNA or tRNA. Then, STAR was used to align the remaining reads to GRCh38, with only uniquely mapping reads retained. PCR duplicates were collapsed based on the unique molecular identifiers and mapping locations. The nucleotide-resolution crosslink position was calculated as the coordinate immediately preceding the reverse transcription truncation event.

### Reverse transcription, qPCR and IR validation

Reverse transcription was performed using the Revert Aid First Strand cDNA Synthesis Kit (ThermoFisher Scientific) using 1 μg of total RNA and random hexamers. qPCR was performed using the PowerUP SYBR Green Master Mix (ThermoFisher Scientific) and the Agilent Mx3000P QPCR System or the QuantStudio 6 Flex Real-Time PCR System (Applied Biosystems). Primers used are listed in Supplementary Table 2. Specific amplification was determined by melt curve analysis and agarose gel electrophoresis of the PCR products. Primer pairs with 90–110% efficiency were used. For each IR validation to screen three primers pairs were used: (1) primer pair F1 R1 (intron spanning, across exon-exon junction) were used to analyse gene expression levels; (2) primer pair F2 R2 (one primer on an exon flanking the intron to be analysed, the other on the intron) was used to assess the levels of IR; and (3) primer pair F3 R2 (both primers on the exons flanking the intron of interest, if possible designed across the exon-exon junction) was used to measure levels of the spliced transcript. RT-minus samples were used as negative controls. Levels of IR (primer pair F2R2) were normalised over the expression level of each individual gene (primer pair F1R1). Gene expression levels were measured using the *ddCt* method using three housekeeping genes (GAPDH, POLR2B and UBE2D3).

### Cell-cycle analysis

Cell-cycle analysis was performed by flow cytometry according to standard protocols. Briefly, cells were dissociated using either Accutase or Trypsin (Life Technologies), washed and fixed in suspension using 70% cold Ethanol. Cells were stained using propidium iodide (PI, 50 μg ml^−1^, Sigma Aldrich) in the presence of RNase A (10 μg/ml, Sigma Aldrich). Cells were analysed using a BD FACS Calibur (BD Bioscience). Doublets were excluded from analysis and 10,000 events were collected in the single-cell gate per sample. Cell-cycle data were analysed using the Multicycle module of FCS Express 6 (De Novo Software).

### Animals, transgenic models and tissue processing

All animal experiments described in this study were carried out under license from the UK Home Office, and were approved by the Ethical Review Panel of the Institute of Neurology. The following transgenic mouse lines were used: (1) *SOD1*^*G93A*^ mice (B6SJL-Tg(SOD1*G93A)1Gur/J, Jackson Laboratories) (2) over-expressing mutant human VCPA232E were generated by J. Paul Taylor et al, St Jude Children’s Research Hospital, Memphis, TN, USA and are described in Custer et al, 2010^69^ and bred to wild-type C57 Black 6 (*C57/B6*) background. Wild-type *C56BL/6-SJL* mixed background (Jackson Laboratories) were used as control. For tissue collection, animals were injected with terminal anaesthesia (pentobarbital sodium, Euthatal) and were transcardially perfused with 4% paraformaldehyde. The lumbar region of the spinal cord was removed and post-fixed with 4% paraformaldehyde and cryoprotected overnight with 30% sucrose; 10 or 20 μm serial transverse cryosections were cut for immunofluorescence staining.

### Human post-mortem tissue

Snap-frozen tissue sections derived from lumbar spinal cords of three age- and sex-matched sporadic ALS patients, disease duration 1, 2 and 2 years, respectively, and three age and gender matched control subjects with no history of neurological disease (*n* = 3 patients and 9 sections; means of ages: 68 + 6.55 and 69.5 + 2.12 years; see Supplementary Table 3). Death to snap-freezing delay times were also comparable between the groups (delay: 25 + 5.29 and 29.33 + 9.29 h, for control and sALS patients, respectively). The spinal cord samples were obtained from the tissue bank NeuroResource, UCL Institute of Neurology, London, UK. Samples were donated to the tissue bank with written tissue donor informed consent following ethical review by the NHS NRES Committee London-Central and stored under a Research Sector Licence from the UK Human Tissue Authority (HTA).

### Immunolabelling and imaging

For immunocytochemistry and immunohistochemistry, samples were blocked in 10% normal goat serum (NGS) or 10% normal donkey serum (NDS) as appropriate and permeabilised in 0.3% Triton X-100 (Sigma-Aldrich; in PBS) at room temperature (RT) for 1 h. Immunolabelling was performed with primary antibodies in NGS (5%) and Triton X-100 (0.1% in PBS) at 4 °C overnight followed by species-specific secondary antibodies for 1 h at RT and DAPI nuclear counterstain (100 ng/ml) for 10 min at RT. For human post-mortem samples fixation and permeabilisation in cold methanol (−20 °C, 20 min) was performed before the immunostaining. Primary antibodies were diluted as follows: rabbit anti Olig2 (Millipore, AB9610) 1:200; mouse anti SMI32 (Cambridge Bioscience, SMI-32R-500) 1:1000; goat anti ChAT (Millipore, AB144P) 1:100; rabbit anti Pax6 (biolegend, 901301) 1:300; mouse and rabbit anti SFPQ (Abcam, ab11825 and ab38148, respectively) 1:100; mouse and rabbit anti beta-tubulinIII (biolegend, 801201), mouse anti Lim3 (DSHB, 67.4E12) 1:50; chicken anti Nkx2.2 (DSHB, 74.5A5) 1:50. Images were acquired using either a 710 Laser Scanning Confocal Microscope (Zeiss) or the Opera Phenix High-Content Screening System (Perkin Elmer). For image analysis, Fiji or the Columbus Image Analysis System (Perkin Elmer) were used.

### Western blotting

Western blotting was performed according to standard protocols (BioRad). Whole cell lysates were obtained from snap-frozen cell pellets using the cOmplete™ Lysis-M EDTA-free lysis buffer (Roche). Protein concentration was determined by Pierce BCA assay (ThermoFisher Scientific) and used to maximise equal loading across the gels (~9 μg per lane). Electrophoresis was run on 4–12% Criterion™ XT Bis-Tris Protein Gel (BioRad) at constant voltage (200 V, one hour) and followed by protein transfer to a nitrocellulose membrane (BioRad). Blocking was performed in PBS, 0.1% Tween, 5% dry milk powder (Marvel) at RT for one hour followed by primary antibody incubation overnight at 4 °C. Primary antibodies were diluted in PBS, 0.1% Tween, 5% dry milk powder as follows: mouse anti SFPQ (Abcam, ab11825) 1:250; rabbit anti TLS/FUS (Abcam, ab84078) 1:500; rabbit anti DDX39 (Sigma Aldrich, SAB2700315) 1:500, rabbit anti DARS (Abcam, ab182157) 1:1000; mouse anti GLN1 (Antibodies-online, AA 1-373) 1:1000; rabbit anti TDP-43 (Abcam, Ab133547) 1:10,000; mouse anti GAPDH (Life Technologies, clone 6C5, AM43000) 1:5000. For detection membranes were incubated with species-specific near infra-red fluorescent antibodies (IRDye, Licor) for one hour at RT and imaged using an Odyssey Fc Imaging System (Licor).

### Data availability

The GEO accession number for the newly generated RNA-seq libraries is GSE98290 (https://www.ncbi.nlm.nih.gov/geo/query/acc.cgi?acc = GSE98290). We also obtained single- and paired-end RNA sequencing reads derived from two independent studies of in vitro neural differentiation of hESCs (GSE20301^22^ and GSE86985^23^); two independent studies on familial forms of ALS either caused by mutant SOD1 (*n* = 5; 2 patient-derived SOD1A4V and 3 isogenic control MN samples where the mutation has been corrected; Hb9 FACS purified MNs, GSE54409^27^ or FUS (*n* = 6; 3 patient-derived FUS R521G and 3 healthy sibling controls MNs, GSE77702^28^; two RNA-seq data from human ESC shared by Miha Modic; two foetal spMN (E-MTAB-3871Gene Expression Omnibus; NIH Roadmap Epigenomics Mapping Consortium; https://www.ebi.ac.uk/arrayexpress/experiments/E-MTAB-3871/) and adult spMN (laser-captured spMN; GSE76514^52,53^) samples. Further information and requests for resources and reagents should be directed to and will be fulfilled by the Lead Contact, Rickie Patani (rickie.patani@ucl.ac.uk).

## Electronic supplementary material

Supplementary Information

Description of Additional Supplementary Files

Supplementary Data 1
