# Supplementary material for: Intron retention and nuclear loss of SFPQ are molecular hallmarks of ALS
Source: Nat Commun. 2018 May 22;9:2010. doi: 10.1038/s41467-018-04373-8 (PMC5964114; doi:10.1038/s41467-018-04373-8)
Supplement: Supplementary file 1 — Supplementary Information [file 41467_2018_4373_MOESM1_ESM.pdf]

Intron retention and nuclear loss of SFPQ are molecular hallmarks of ALS

Luisier*, Tyzack*, Hall, Mitchell, Devine, Taha, Malik, Meyer, Greensmith, Newcombe, Ule, Luscombe, Patani

Supplementary Information

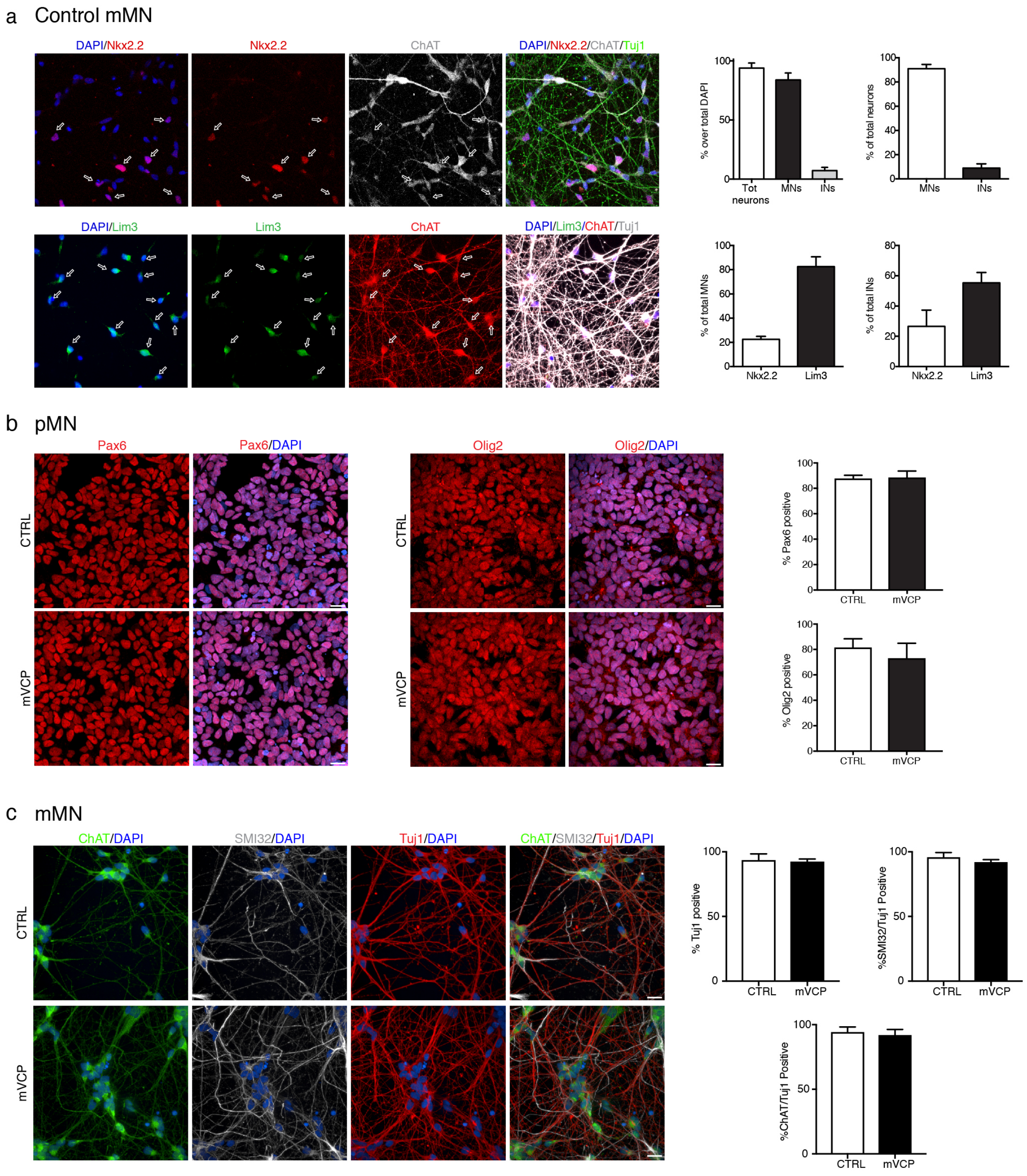

Supplementary Figure 1. Characterisation of iPSC derived cultures.
(a) After 17 days of terminal differentiation, both control and *VCP*^{mu} mMNs were immunolabeled for β-tubulin III to identify all neurons, ChAT to label MNs and either NKX2.2 (*top panels*) or Lim3 (*bottom panels*) to determine neuronal subtype identity. Interneurons (INs) were defined as β-tubulin III positive/ChAT negative cells. Graphs show: the proportion of neurons, MNs and INs over the total number of cells; the relative proportion of MNs and INs of all neurons (i.e. of all β-tubulin III positive cells); the proportion of Nkx2.2 (V3) and Lim3 (V2) positive INs respectively. Data is expressed as mean ± SEM from ≥ 4 line. **(b)** To determine the efficiency of ventral spinal cord specification of motor neuron precursors (pMNs), both control and *VCP*^{mu} pMNs were immunolabelled for the pMN domain markers Pax6 (*left panel*) and Olig2 (*right panel*). Graphs show the proportion of Pax6 or Olig2 positive cells over the total number of cells (i.e. over number of DAPI counterstained nuclei). Data is expressed as mean ± SEM from 3 control lines and 4 *VCP*^{mu} lines. **(c)** The efficiency of mature motor neuron (mMN) specification from control compared to *VCP*^{mu} cultures was assessed by immunocytochemistry, analysing the number of mMNs (i.e. ChAT and SMI32 positive cells) over the total number of neurons (i.e. of all β-tubulin III positive cells). The proportion of β-tubulin III positive cells over the total number of cells was used to compare the efficiency of neuronal differentiation. Data is mean ± SEM from 3 control lines and 3 VCP lines. Scale bar in (**a**), (**b**) and (**c**) is 20μm.

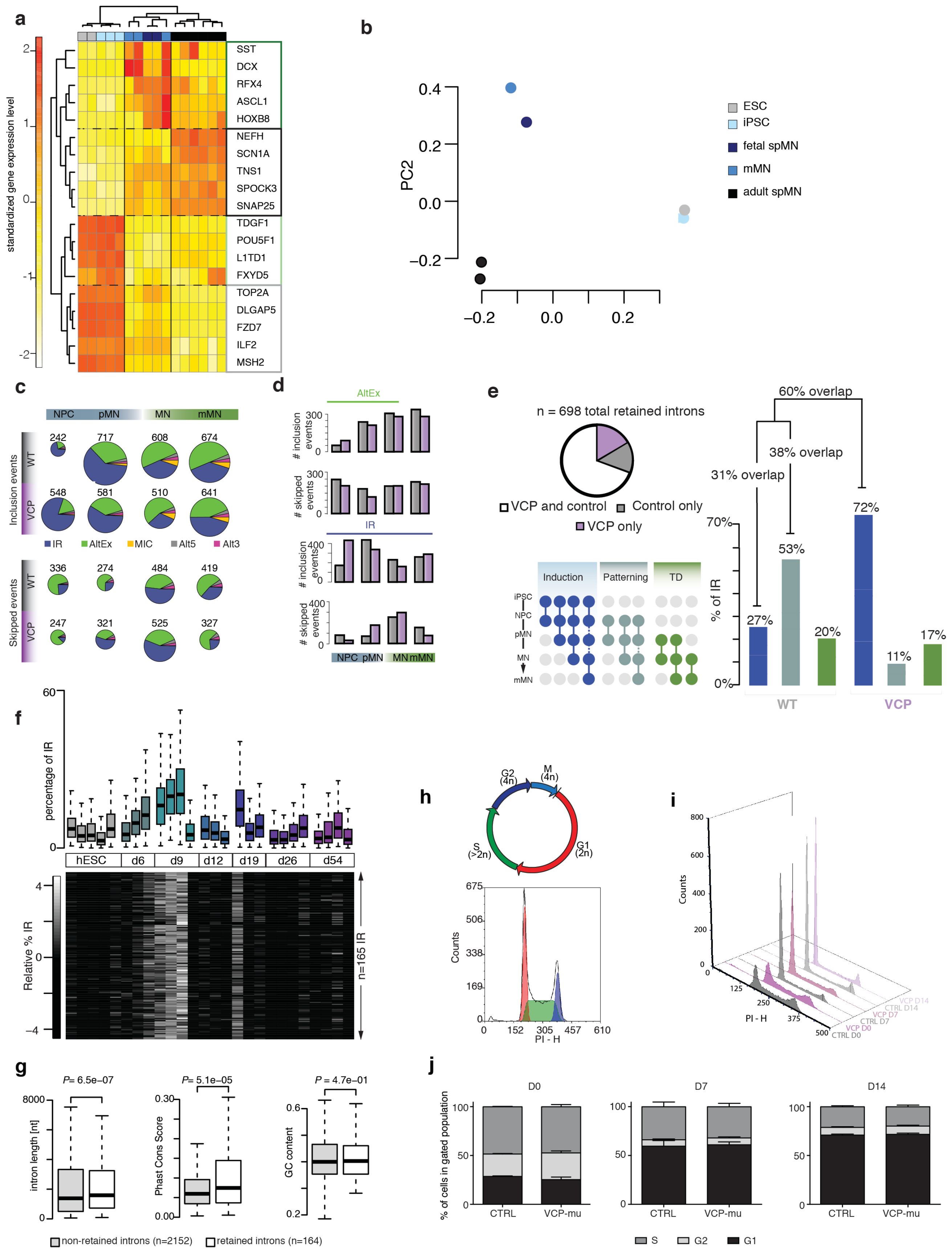

Supplementary Figure 2

(**a**) Heatmap of the standardized expression of 19 key gene markers of spinal MN (spMN) maturation and embryonic development identified in a previous study [20]. Samples in green come from the present study. Two ESC RNA-seq datasets were obtained from collaborators (unpublished data). Two foetal spMN [52]NIH Roadmap Epigenomics Mapping

Consortium[52] and two adult spMN (laser-captured spMN; [53]) samples were downloaded from Array Express and GEO. (**b**) PCA performed on normalized gene expression values of key gene markers. Samples are plotted by their coordinates along PC1 and PC2. Colors of data points indicate similar sample types. ESC (grey), iPSC (light blue), mMN (blue), fetal spMN (navy) and adult spMN (black). **(c)** Pie charts representing distributions of regulated included (*upper*) and skipped (*lower*) splicing events in healthy control and *VCP*[*mu*] samples at distinct stages of motor neurogenesis compared to iPSCs or previous time-point. Intron retention (IR); alternative exon (AltEx); micro-exons (MIC); alternative 5' and 3' UTR (Alt5 and Alt3). **(d)** Bar graphs representing the number of included (*upper*) and skipped (*lower*) exonic and intronic splicing events respectively in healthy controls (grey bars) and *VCP*[*mu*] samples (magenta bars) at specific time points during MN differentiation. (**e**) (*upper left*) Pie chart representing percentages of retained introns across all time points that are either shared between *VCP*[*mu*] and control samples (white) or specific to either control (grey) or *VCP*[*mu*] samples (magenta). (*lower left*) Schematic showing colour codes for different categories of IR grouped according to stage of initiation and persistence. Coloured circles indicate sample status and line between circles indicate event statistically significant between sample and either prior sample from prior status or iPSC. (*right*) Bar graphs representing the percentage of IR events upon induction, patterning or terminal differentiation in healthy control or VCP samples. The height of the different coloured stacked bars represents the relative number of IR events that last until specific stages of lineage restriction as shown on lower left part of the panel. Percentage of overlap between events in control and *VCP*[*mu*] events is shown above the stacked bars. (**f**) Percentage of retention for 167 manually curated introns in a study of in vitro neural differentiation of hESCs for 54 days [23]. (**g**) Comparison of intron lengths, conservation scores and % GC of retained (compared to non-retained) intron sequences of the high confidence set of 167 genes. Data shown as box plots in which the centre line is the median, limits are the interquartile range and whiskers are the minimum and maximum. *P*-values obtained from Welch test. **(h)** Diagram showing cell cycle and its analysis. DNA content of each cell is quantified by measuring the intensity of propidium iodide (PI) staining by flow cytometry. Cells in G2 (tetraploid, 4n, blue) have a two times higher PI intensity than cells in G1 (diploid, 2n, red). Cells in the S phase are replicating their genetic material and have intermediate PI intensity (>2n, green). A representative flow cytometry histogram with automated interpolation of each phase is shown on the right. (**i**) Representative cell cycle histograms for both control and *VCP*[*mu*] lines at each time point. (**j**) At each time point the proportion of cells in either G1 (black), S (grey) or G2/M (light grey) were automatically calculated. Data is expressed as mean+SD, N=4 control lines vs N=4 *VCP*[*mu*] lines from two independent experiments (i.e. two separate neural inductions per line).

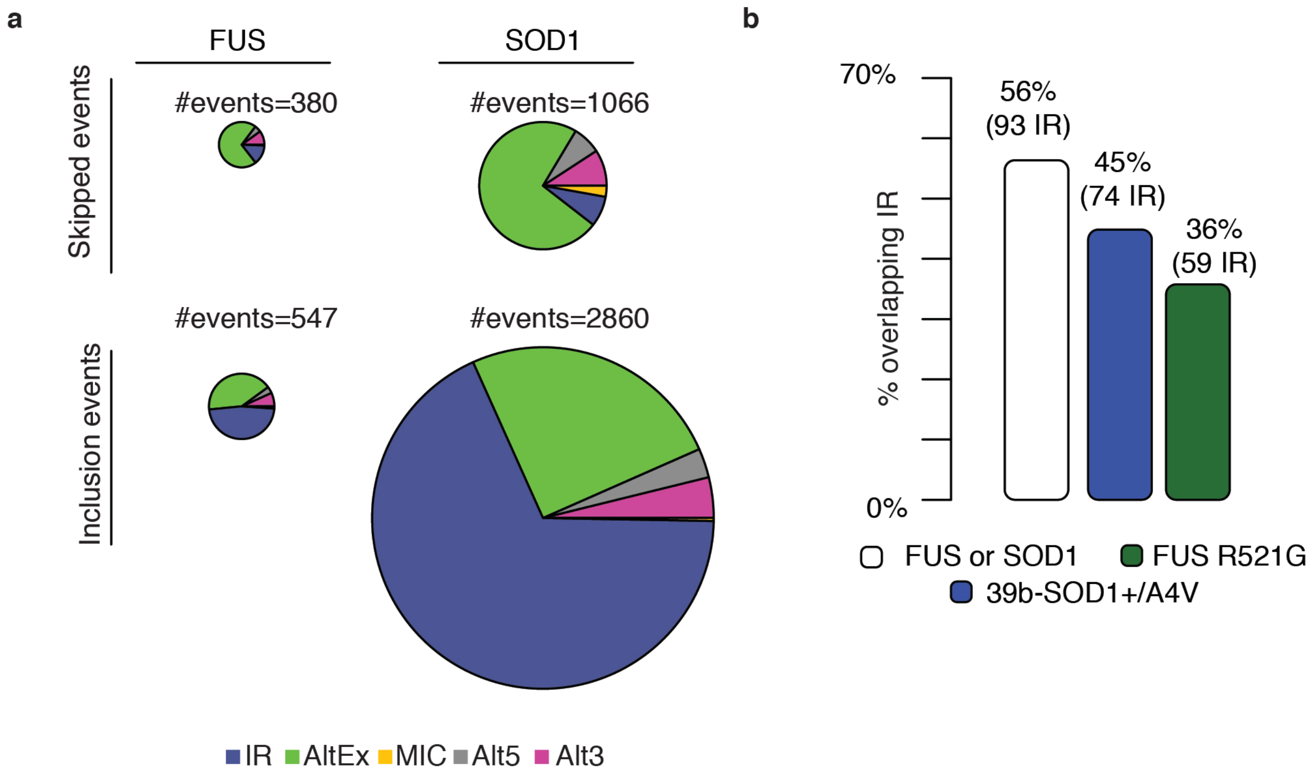

Supplementary Figure 3

(a) Pie charts representing distributions of regulated splicing events in SOD1 and FUS mutant MNs compared to controls. Intron retention (IR); alternative exon (AltEx); micro-exons (MIC); alternative 5' and 3' UTR (Alt5 and Alt3). **(b)** Bar graphs depicting the percentage of retained introns upon MN differentiation identified in this study (167 events in 143 genes) that also exhibit significant retention in MN samples harbouring either SOD1 or FUS gene mutations (white bar), in *SOD1^{mu}* MNs (blue bar) or *FUS^{mu}* MNs (grey bar) [28,50].

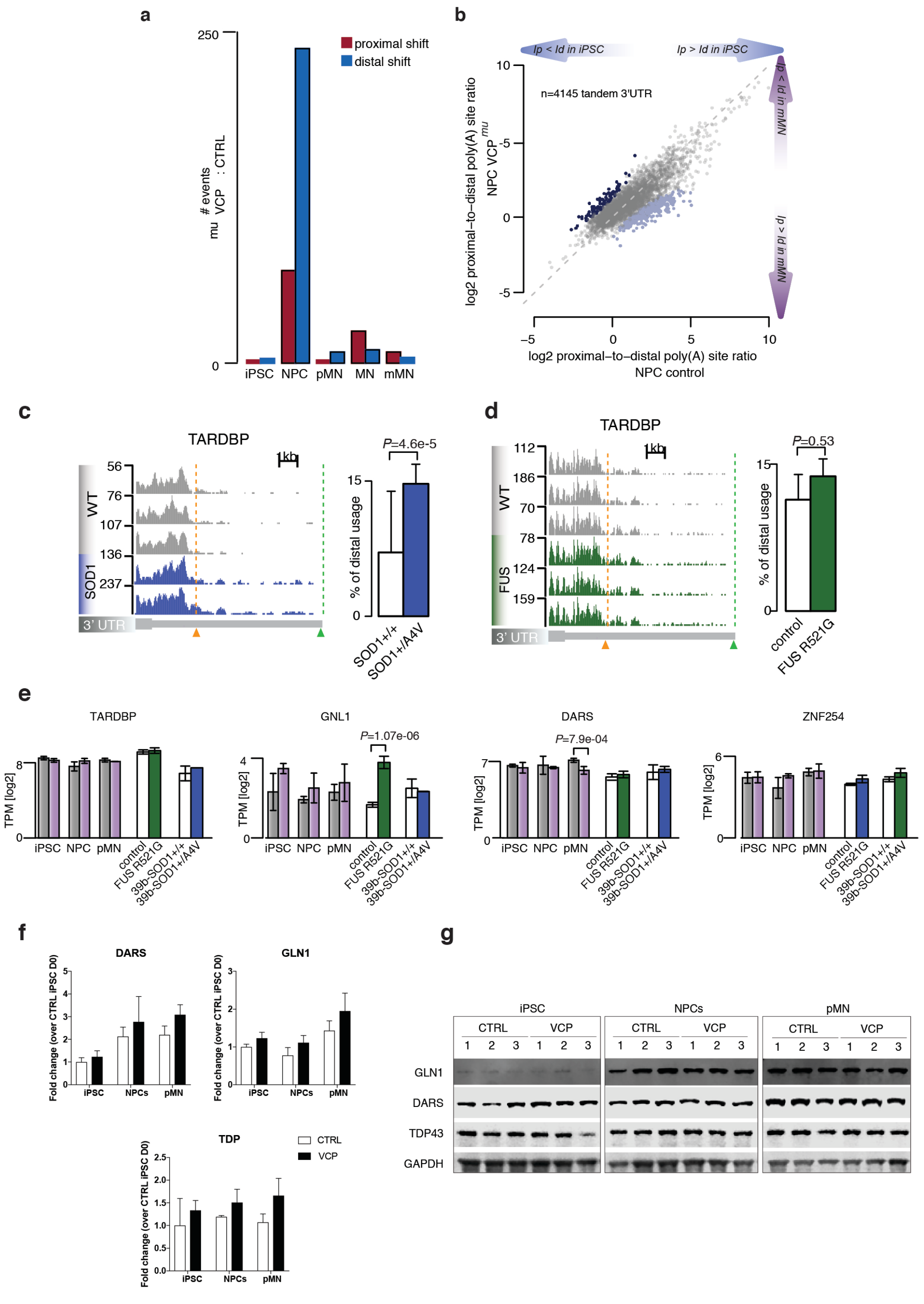

Supplementary Figure 4

(**a**) Bar plot showing number of 3' UTR with significant promoter-proximal (*red*) and promoter-distal (*blue*) shifts at each stage of differentiation in *VCP*^{mu} samples compared to control samples.(**b**) Scatter plot of the relative use of promoter-proximal and promoter-distal poly(A) sites in control and *VCP*^{mu} samples at NPC stage. FDR<0.01 between *VCP*^{mu} and control NPC samples (Fisher exact test). Dark blue = promoter-proximal shifts in VCP^{mu} compared to control. Light blue = promoter-distal shifts in *VCP*^{mu} compared to control. (**c** and **d**) (*Left*) Genome browser view of the 3' UTR of the gene TARDBP exhibiting significant shift towards increased promoter-distal poly(A) site usage in SOD1^{mu} MN compared to control, but not in *FUS*^{mu}. (*Right*) Bar plots showing the distal 3' UTR usage relative to short 3' UTR. *P*-value obtained from Fisher count test. (**e**) TARDBP, GNL1, DARS and ZNF254 gene level quantification as obtained by Kallisto (Bray et al. 2016). *P*-Value as obtained by differential gene expression analysis with Sleuth (Pimentel et al. 2016). (**f**) Gene expression levels of GLN1, TARDBP and DARS measured by qPCR and normalised over expression levels in CTRL iPSC. GAPDH, POLR2B and UBE2 were used as housekeeping genes. (**g**) Western blot showing comparable protein levels of GLN1, TDP43 and DARS in control and VCP^{mu} at any given time point. Samples from 3 control and 3 *VCP*^{mu} lines are shown.

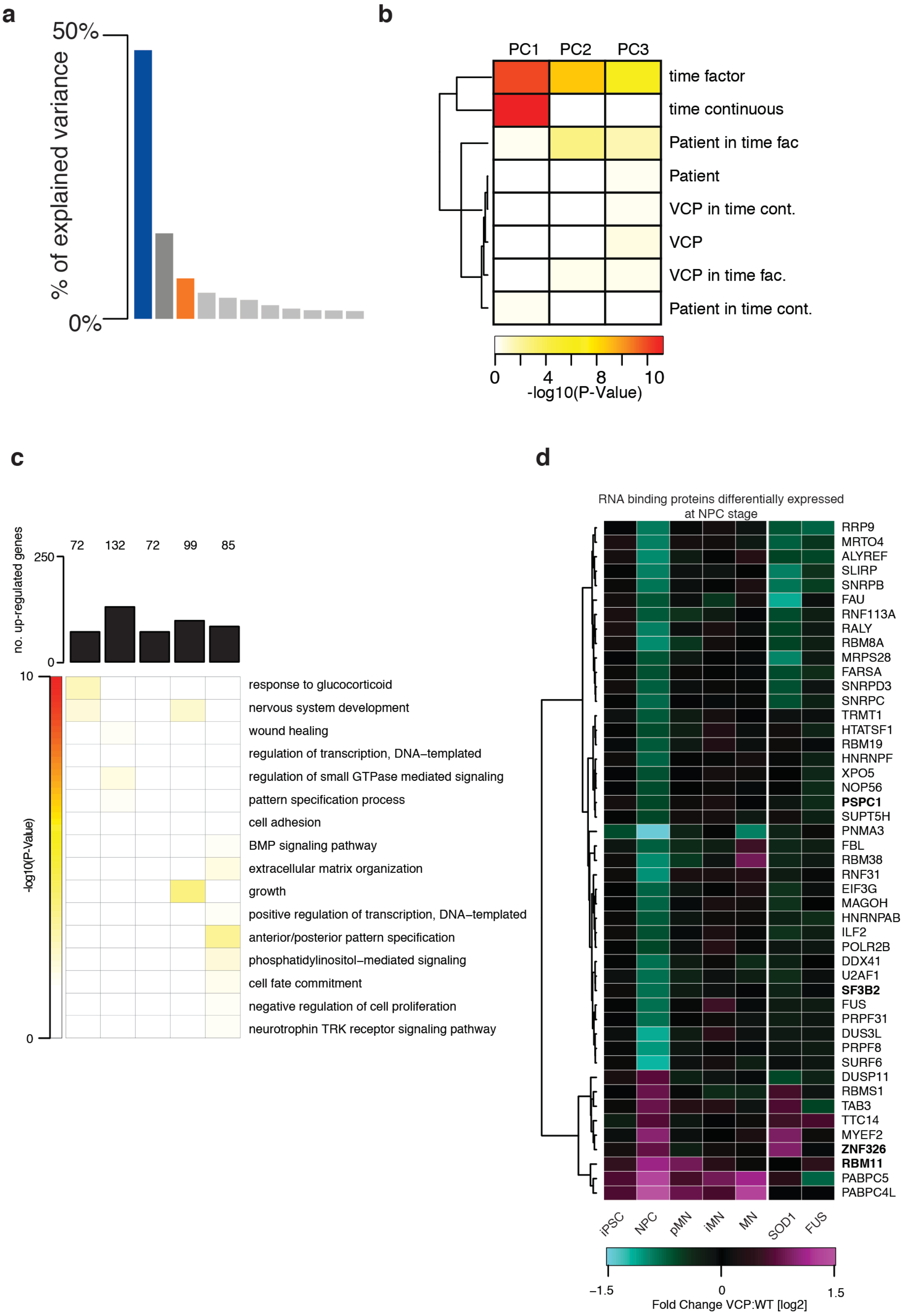

Supplementary Figure 5

(a) Fraction of variance in gene expression captured by the corresponding principal components. **(b)** Heatmap of the significance of association between each variable in this study and the left singular vectors of the components 1, 2 and 3. Significance was obtained from P-value of the multivariate linear model. **(c)** (*upper*) Number of up-regulated genes in *VCP*^{mu} compared to control at distinct stages of MN differentiation shown as bar plot. (*lower*) Heatmap of the biological terms enriched in up-regulated genes at corresponding time-point. Colour-scale = -log10(P-Value) of GO enrichment. **(d)** Log2 fold change of 44 significantly differentially expressed RBPs between *VCP*^{mu} mutant and controls. Colour-scale= log2 fold-change between mutant and controls.

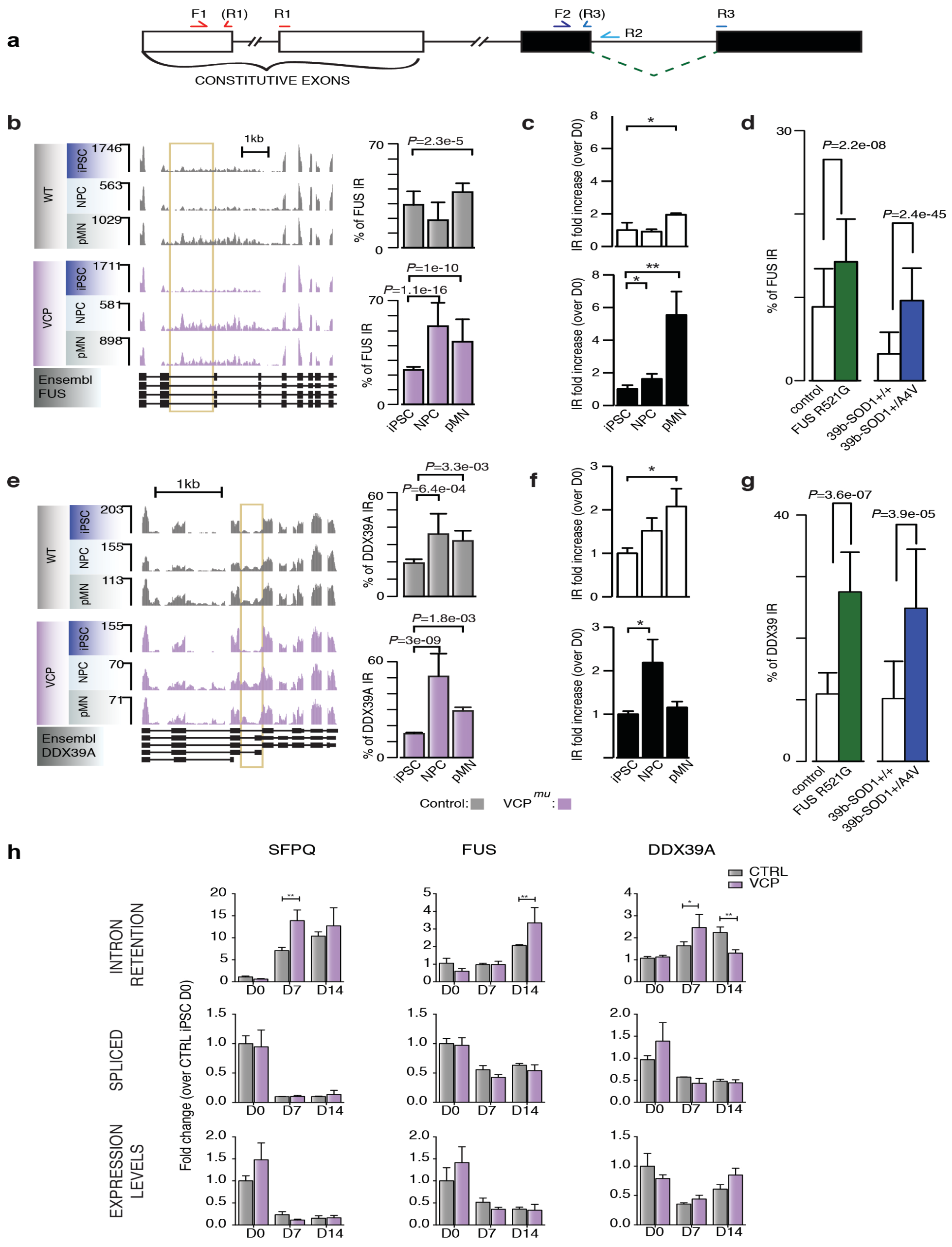

Supplementary Figure 6.

(**a**) Experimental design for qPCR validation of intron retention. For each transcript to be analysed, three primer pairs were used. Primer pair F1 R1 (intron spanning, across exon-exon junction) were used to analyse gene expression levels. Primer pair F2 R2 (one primer on an exon flanking the intron to be analysed, the other on the intron) was used to assess the levels of intron retention. Primer pair F2 R3 (both primers on the exons flanking the intron of interest, if possible designed across the exon-exon junction) was used to measure levels of the spliced transcript. (**b**) (*left*) Visualization of the RNA-seq read profiles of the intron-retaining gene FUS in control and *VCP*^{mu} samples at iPSC, NPC and pMN stages. Intron of interest is indicated with yellow bar. (*right*) Bar graphs quantifying percentage intron retention across the entire time-course in controls and VCP samples (mean ± SD; Fisher count test). (**c**) IR levels measured by qPCR. Levels of IR were measured using primer pair F2R2 and were normalised over the gene's expression levels (primer pair F1R1). To evaluate changes in the levels of IR over time within a group, IR levels at every time point are directly compared to those at the iPSC stage of the same group. N=3 control lines and N=4 VCP lines (mean+SD. *p<0.05, **p<0.01, one-way ANOVA with Dunnet correction for multiple comparisons). (**d**) Percentage intron retention in control compared with *FUS*^{mu} or *SOD1*^{mu} MNs for the gene FUS (mean ± SD; Fisher count test).(**e**) Same as **b** for DDX39 gene. (**f**) Same as **c** for DDX39 gene. (**g**) Same as **d** for DDX39 gene. (**h**) IR levels measured by qPCR. Levels of IR were measured using primer pair F2R2 and were normalised over the gene's expression levels (primer pair F1R1). Gene expression levels were normalised using three housekeeping genes (GAPDH, POLR2B and UBE2D3). To compare levels of IR at any given time point across groups, IR levels were normalised over IR levels in control cells at D0. N=3 control lines and N=4 VCP lines. Data is expressed as mean+SD. *p<0.05, **p<0.01, two-way ANOVA with Sidak's correction for multiple comparisons.

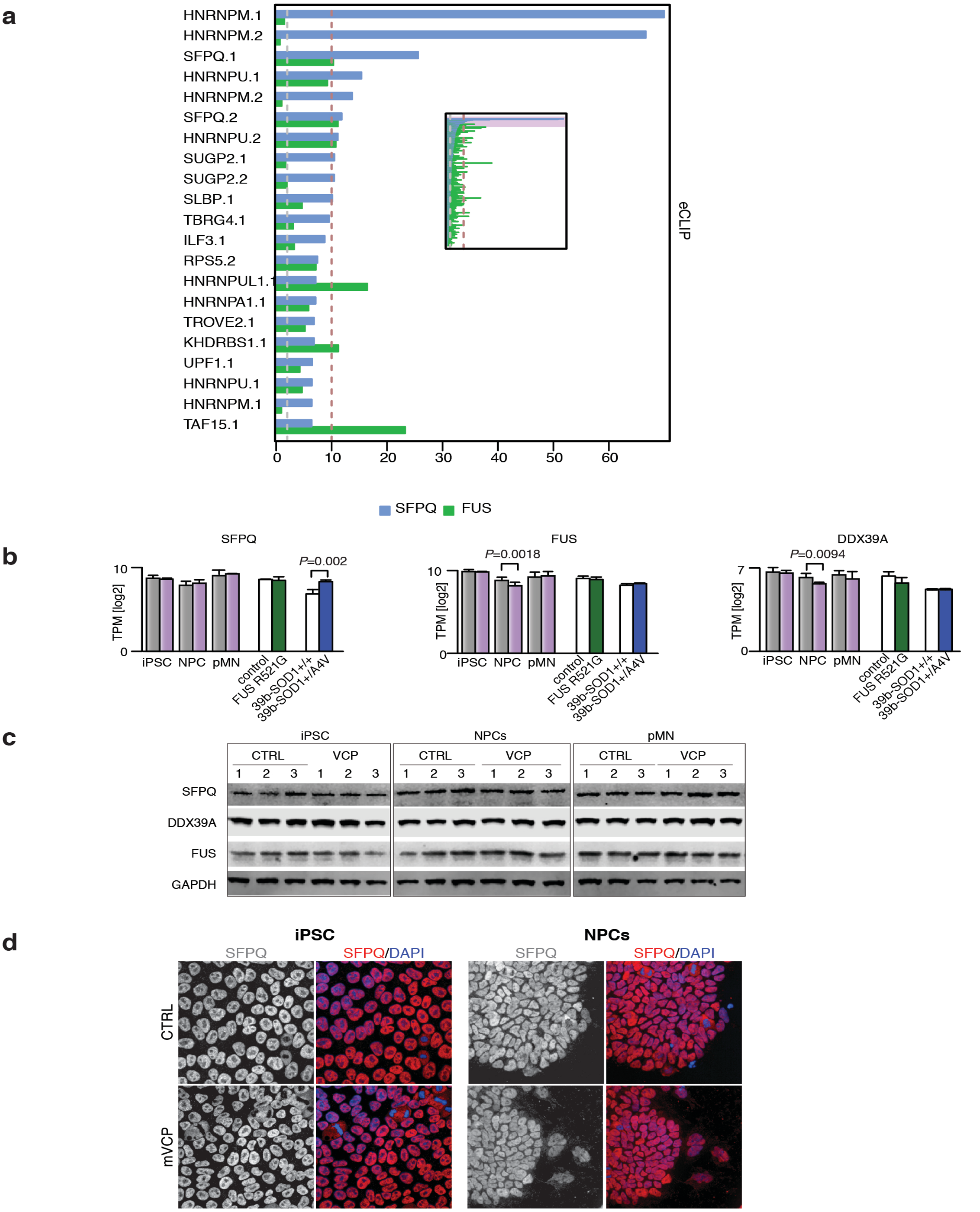

Supplementary Figure 7

(**a**) Enrichment of RBPs crosslink events mapping to the retained intron compared to non-retained introns within corresponding genes genes; blue bars = SFPQ gene; green bars = FUS gene. (**b**) SFPQ gene level quantification as obtained by Kallisto [54]. P-Value as obtained by differential gene expression analysis with Sleuth [55].(**c**) Western blot showing comparable protein levels of SFPQ, FUS and DDX39 in control and *VCP*^{mu} at iPSC, NPC and pMN stage. Samples from 3 control and 3 *VCP*^{mu} lines are shown.(**d**) Representative images showing subcellular localization of SPFQ determined by immunocytochemistry in iPSCs, NPCs and pMNs. Scale bar is 20μm.

Supplementary Table 1. iPSC lines used in this study.

iPSC line	Mutation present	Age of Donor	Age at disease onset	Sex of Donor
CTRL 1	None	78	N/A	Male
CTRL 2	None	64	N/A	Male
CTRL 3	None	(unknown)	N/A	Female
MUT 1	R155C	43	40	Female
MUT 2	R155C	43	40	Female
MUT 3	R191Q	42	36	Male
MUT 4	R191Q	42	36	Male

Supplementary Table 2. List of primers used.

Region	Primer
SFPQ F1	GCCGAATGGGCTACATGGAT
SFPQ R1	TCAGTACGCATGTCACTTCCC
SFPQ F2	GTGGATCGACTCATTGGTGA
SFPQ R2	TTCCTCTAGGACCCTGTCCA
SFPQ F3	GATGGGAAGTGACATGCGTA
FUS F1	GGAAGTGTCCTAATCCCACCT
FUS R1	TAGGGGCCTTACACTGGTTG
FUS F2	AGCAGTGGTGGCTATGAACC
FUS R2	GCACTAGGGACTGGCTTCAG
FUS R3	GGGCCACCAAATTTATTGAA
DDX39A F1	GCAGATTGAGCCTGTCAACG
DDX39A R1	ACACAGACACCTTGACGCTG
DDX39A F2	AGGTGTTTGTGGACGACGA
DDX39A R2	GAGCTTGCGGTTCTTCTCAC
DDX39A F3	CAGGATCCCATGGAGGTG
GAPDH F	ATGACATCAAGAAGGTGGTG
GAPDH R	CATACCAGGAAATGAGCTTG
POLR2B F	CAGAAAAGGTTCTGATTGCCCAAGA
POLR2B R	AATGCGCTGACCAATAGCAC
UBE2D3 F	GCCCATATCAAGGCGGTGTA
UBE2D3 R	CAGGCGACCACTGTGATCTT
GLN1 F	GCCATCTTCCCTCCCAGTATC
GLN1 R	TCACAATTGGGGTGGCAGAG
DARS F	TGCACAGGCTGTACTTTAGGT
DARS R	TCCTACTGATGCATGTCTGAAGT
TARDBP F	GACGGTGGGTGTCCCATTTT
TARDBP R	ACTAGCCTTTCATGCCTCGG

Supplementary Table 3. Information of the patients used in the post mortem tissue analysis.

Age	Gender	Category	Cause of death	Post mortem delay (hours)
71	M	Control	Burst aortic aneurysm	25
68	F	Control	Colorectal metastatic cancer	23
68	M	Control	Heart disease	40
69	M	sALS	MND	19
61	M	sALS	MND	29
74	F	sALS	MND	27
