# Supplementary material for: Intron retention and nuclear loss of SFPQ are molecular hallmarks of ALS
Source: Nat Commun. 2018 May 22;9:2010. doi: 10.1038/s41467-018-04373-8 (PMC5964114; doi:10.1038/s41467-018-04373-8)
Supplement: Supplementary file 2 — Description of Additional Supplementary Files [file 41467_2018_4373_MOESM2_ESM.pdf]

Description of Additional Supplementary Files

File Name: Supplementary Data 1
Description: Metadata table of the RNA-seq samples generated in this study.
